# A Roadmap to Transform Lung Cancer Outcomes: Priorities in Biology, Therapeutic Innovation, Early Detection, Prevention and Interception

**DOI:** 10.1158/2159-8290.CD-25-2318

**Published:** 2026-06-01

**Authors:** Monte M. Winslow, Mohamed A. Ahmed, Christine D. Berg, James R. M. Black, Julian Downward, Ramaswamy Govindan, Roy S. Herbst, John V. Heymach, Elizabeth M. Jaffee, Norbert Kraut, Miriam Merad, Matthew Meyerson, Tej Pandya, Katerina Politi, Arati V. Rao, Charles M. Rudin, Jean-Charles Soria, Yuning J. Tang, Kwok-Kin Wong, Timothy A. Yap, Charles Swanton

**Affiliations:** 1 Department of Genetics, Stanford University School of Medicine, Stanford, CA, USA; 2 Department of Pathology, Stanford University School of Medicine, Stanford, CA, USA; 3 American Association for Cancer Research, Philadelphia, PA, USA; 4 Early Cancer Detection Consultant; NCI (retired), Bethesda, MD, USA; 5 Cancer Evolution and Genome Instability Laboratory, The Francis Crick Institute, London, UK; 6 Oncogene Biology Laboratory, Francis Crick Institute, London, UK; 7 Division of Oncology, Washington University, St. Louis, MO, USA; 8 Yale Cancer Center, Yale University School of Medicine, Smilow Cancer Hospital, New Haven, CT, USA; 9 Department of Thoracic/Head and Neck Medical Oncology, The University of Texas MD Anderson Cancer Center, Houston, TX, USA; 10 Sidney Kimmel Comprehensive Cancer Center, Johns Hopkins University, Baltimore, MD, USA; 11 Discovery Research, Boehringer Ingelheim RCV GmbH & Co KG, Vienna, Austria; 12 Department of Immunology and Immunotherapy, Precision Immunology Institute, Icahn School of Medicine at Mount Sinai, New York, NY, USA; 13 Department of Medical Oncology, Dana-Farber Cancer Institute, Boston, MA, USA; 14 Departments of Genetics and Medicine, Harvard Medical School, Boston, MA, USA; 15 UKRI UCL Centre for Doctoral Training in AI-enabled Healthcare Systems, University College London, London, UK; 16 Departments of Pathology and Internal Medicine & Yale Cancer Center, Yale School of Medicine, New Haven, CT, USA; 17 Thoracic Oncology Development Head, Pfizer Inc. New York, NY, USA; 18 Department of Medicine, Memorial Sloan Kettering Cancer Center, New York, NY, USA; 19 R&D, Amgen Inc., Thousand Oaks, CA, USA; 20 Laura and Isaac Perlmutter Cancer Center, New York University Langone Health, New York, NY, USA; 21 Therapeutics Discovery Division, The University of Texas MD Anderson Cancer Center, Houston, TX, USA; 22 Cancer Research UK Lung Cancer Centre of Excellence, University College London Cancer Institute, London, UK; 23 Department of Medical Oncology, University College London Hospitals, London, UK

## Abstract

Advances in targeted therapies, immunotherapy, and early detection have revolutionized lung cancer treatment and extended survival. Nonetheless, lung cancer remains highly fatal. Here, we identify knowledge gaps and propose critical areas of future research, aligning with the mission of the AACR Lung Cancer Task Force. We delineate research priorities, including advancing prevention initiatives, enhancing early detection strategies, developing novel treatments, and refining patient stratification. Addressing disparities and increasing efforts on relatively neglected lung cancer subtypes are also essential. Finally, international collaboration, centralized clinical trial databases, novel clinical trial designs, and artificial intelligence-driven analytics should accelerate precision medicine and aid in elucidating drug resistance mechanisms. Together, these efforts promise to improve patient outcomes.

## Introduction

Lung cancer is a constellation of diverse cancer types that together comprise a major cause of cancer-related mortality worldwide, collectively accounting for an estimated 1.8 million deaths per year (1). Despite notable advances in targeted therapies, immunotherapies, and early detection methods, overall survival rates remain lower than many other cancers, which is largely due to late-stage diagnosis and limited curative treatment options (1).

**Figure (1):**
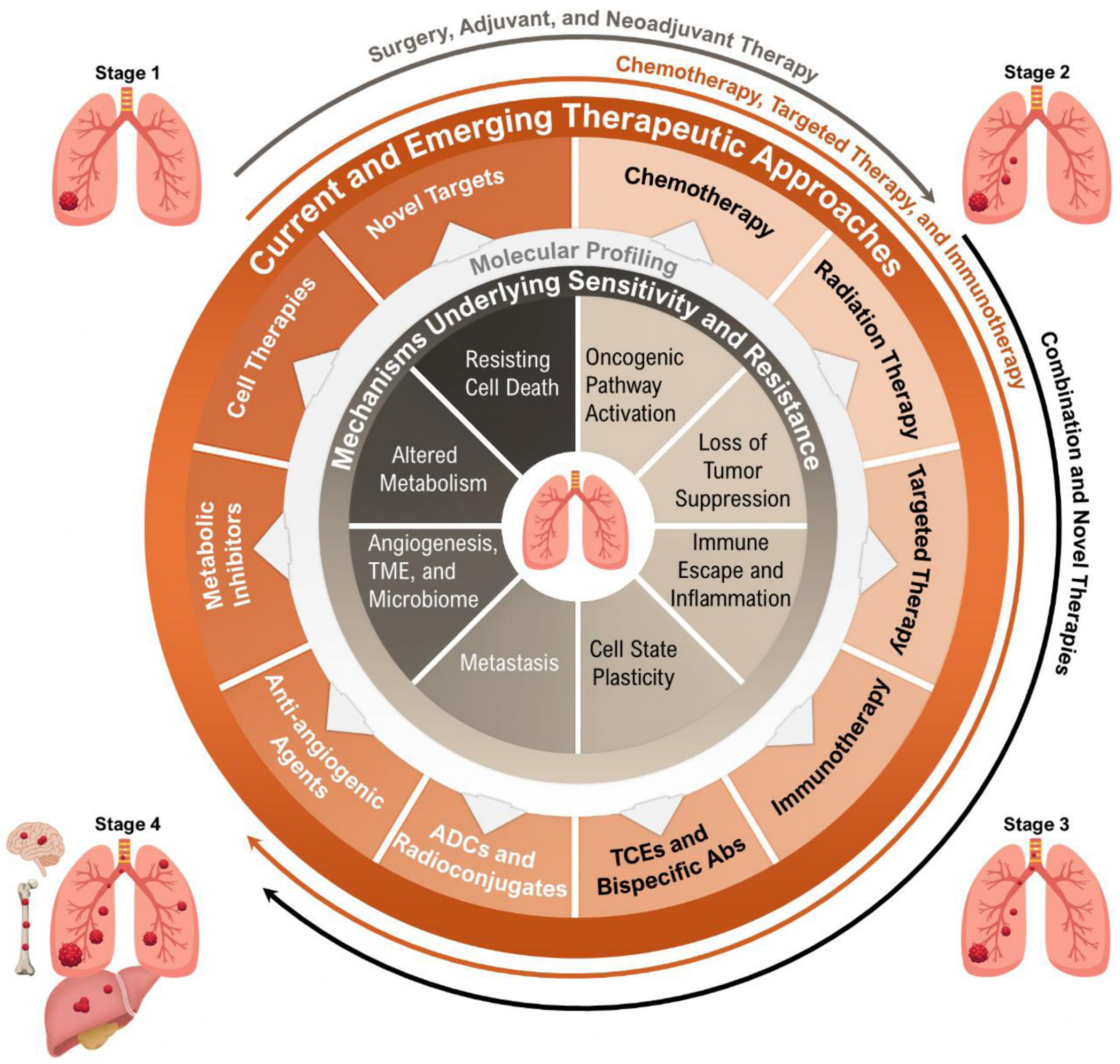
Hallmarks of lung cancer. The hallmarks depicted illustrate how diverse molecular, cellular, and microenvironmental changes underlie sensitivity and resistance to therapies, emphasizing the critical importance of molecular profiling in lung cancer, as well as current and future therapeutic approaches. Tumorigenic processes evolve dynamically across disease stages, from early lesions shaped by environmental injury and inflammation to invasive and metastatic disease sustained by profound genomic, metabolic, and microenvironmental reprogramming. The inner circle depicts key mechanisms underlying tumor sensitivity and resistance, including cell-intrinsic changes such as oncogenic pathway activation (e.g., EGFR and KRAS mutations, and ALK translocations), loss of tumor-suppressor function (e.g., TP53 inactivation), and immune escape, as well as changes in the microbiome, immune environment, tumor microenvironment (TME), and the presence of metastatic disease. The outer circle illustrates current and emerging therapeutic approaches for lung cancer, including chemotherapy, radiation, targeted therapies (e.g., EGFR, KRAS, and ALK inhibitors), and immunotherapy (e.g., PD-1/PD-L1 inhibitors), as well as ADCs, T-cell engagers (TCE), metabolic inhibitors, and novel targets. (Adapted with permission from the figure originally published in ref. (158)).

Lung cancer is unique in its global burden, biological complexity, and strong environmental links. Discoveries in lung cancer have defined fundamental principles of carcinogenesis, tumor evolution and clonal selection; pioneered targeted therapies and immunotherapies; and provided some of the most definitive epidemiological evidence linking exposures such as tobacco smoke and air pollution to cancer risk (Fig. 1). Lung cancer has also served as a proving ground for innovations in cancer therapy: targeted therapies against EGFR, ALK, and KRAS have transformed clinical outcomes; immunotherapies have redefined treatment paradigms; and antibody drug conjugates, bispecific antibodies, T-cell engagers, and cellular therapies are expanding the therapeutic arsenal. Advances in clinical trial design, biomarker-led precision medicine, and liquid biopsy technologies, many originating in lung cancer research, now inform cancer management more broadly. For these reasons, lung cancer should continue to be recognized not only as a global health priority, but also as a cornerstone for scientific and clinical discovery, exemplifying how deep biological understanding can be translated into durable therapeutic benefit. The AACR Lung Cancer Task Force is responding to this ongoing challenge by defining several important research priorities, aimed at prevention, early detection, overcoming therapy resistance, and accelerating progress toward a cure.

## Basic and Translational Research On Mechanisms of Lung Tumorigenesis

Insights into lung cancer biology and the translation of these findings to the clinic, especially over the past two decades, have transformed our understanding of the disease and directly contributed to improved patient outcomes. Continued progress along this trajectory will require a deeper understanding of how cancers progress and evolve, including through treatment. Given the importance of the tumor micro- and macro-environment in shaping lung tumor progression and treatment response, emerging single-cell and spatial profiling approaches coupled with improved *in vivo* models that accurately recapitulate disease biology, including metastasis and immunotherapy response, will be critical for these efforts. Additionally, while great strides have been made in leveraging tumor genetics for therapeutic and diagnostic purposes, a holistic understanding of the interplay between genomic alterations and other key molecular determinants that drive cellular plasticity and define the overall molecular “wiring” of tumors remain elusive. In particular, advancing our understanding of the cancer epigenome and proteome, and integrating these data with tumor genetics and transcriptomics, will be critical for identifying new vulnerabilities in lung cancer, especially at the earliest stages of tumor initiation. This will be especially important for cancers lacking clear actionable oncogenic driver alterations and for treatment-refractory disease.

### Cancer Heterogeneity and Evolution

Lung cancer evolutionary studies have illuminated how intratumor heterogeneity and branched Darwinian evolution shape disease progression, treatment response, and clinical outcomes. Longitudinal multi-region, and multi-omics profiling of early- and late-stage non-small cell lung cancer (NSCLC) have shown that the clonal architecture of a treatment naïve tumor can predict both the timing and pattern of relapse and survival outcome (2,3). Furthermore, chromosomal instability is associated with distinct routes of metastasis with a predilection for extrathoracic metastatic spread and drives immune evasion through HLA and clonal neoantigen loss (2,4,5). Studies have also defined mutational signatures of environmental exposure, shown how chromosomal instability fuels diversification, and pioneered tumor informed ctDNA tracking to capture minimal residual disease and evolutionary dynamics in real time (6,7). Collectively, these insights have established lung cancer as a paradigm for studying cancer evolution and set a global benchmark for integrated longitudinal cancer research.

### Interactions with the Micro- and Immune-Environment

Lung tumor initiation and progression are critically shaped by reciprocal interactions among epithelial and neoplastic cells, the surrounding stroma, and the adaptive and innate immune microenvironment. In lung cancer, inflammatory cues from both intrinsic and extrinsic sources, such as particulate matter exposure, aging, infection, and clonal hematopoiesis, may create a permissive niche that supports the origins, survival, and potentially metastatic dissemination of oncogenically primed progenitor cells (8,9). Myeloid-derived cytokines can drive alveolar progenitors into transitional KRT8^+^ states with impaired differentiation and increased plasticity, likely involving profound epigenetic changes driven by the combination of microenvironment-induced inflammation and an oncogenic mutation in the progenitor population (10). Meanwhile, stromal fibroblasts and endothelial cells remodel the extracellular matrix and provide metabolic and paracrine support that may further enhance cancer cell fitness (11).

As tumors evolve, cancer cell/immune crosstalk becomes increasingly dynamic, driving local immunosuppression, macrophage reprogramming, and T-cell exclusion, while cancer-intrinsic pathways may modulate cytokine release and influence antigen presentation. Spatial and single-cell analyses are beginning to reveal that these ecological relationships evolve in tandem with tumor phylogenies, impacting clonal selection (with evidence of immunoediting of tumors as they evolve), enhancing immune evasion and metastatic potential (12).

Beyond the local microenvironment, tumors engage with the systemic environment through neural, endocrine, and metabolic pathways. For example, it is increasingly appreciated that secreted factors such as GDF15 contribute to cachexia (13). An active area of investigation is how and whether neural signals emanating from the tumor to the CNS may contribute to fatigue and mood disturbances, and how peripheral and central nervous system circuits might modulate immunity, metabolism, and stress responses that, in turn, influence tumor behavior (13). Understanding reciprocal tumor-host interactions, from the lung to the CNS/PNS axis, is an exciting area of research that may help to define how systemic physiology both constrains and enables lung cancer progression.

Future efforts should focus on dissecting the temporal sequence of these interactions to identify actionable targets to intercept the earliest steps in tumor initiation, define how early inflammatory and stromal states establish the ecosystem in which malignant clones emerge, and how tumor evolution subsequently reshapes that environment to sustain growth and resist immune clearance.

### Advancing Biological Models

Models of lung cancer have been at the forefront of cancer modeling, capitalizing on novel methods for genome engineering (14–16) and *ex vivo* culturing (17), as well as integration with emerging high-throughput molecular profiling technologies (18). Preclinical models that recapitulate key aspects of lung carcinogenesis are imperative for studying the biology of these tumors and testing new therapies. Continued advances in diverse model systems that faithfully recapitulate the human disease, and integrating these models with clinical datasets, will be essential for progress in understanding the mechanisms of lung cancer development and response to therapy.

Most existing genetically engineered mouse models rely on gain- or loss-of-function mutations in one or two driver genes (19). In contrast, human tumors are both genomically diverse and complex, often harboring multiple driver mutations and structural chromosomal alterations. Consequently, there is a need to develop more genetically complex lung cancer mouse models. Continued advances in technologies that enable the simultaneous perturbation of multiple genes and modeling of a wider range of somatic events, including translocations and extrachromosomal DNA (20,21), will lead to better models that more accurately reflect the full spectrum of genomic alterations in human lung tumors. Moreover, most genetically engineered mouse models do not readily develop metastatic disease. Given that metastatic disease is a major barrier to disease control, there is an urgent need to generate better *in vivo* metastatic models that permit mechanistic studies of metastatic progression and more accurate analyses of therapeutic response in the metastatic context (22).

The variable success of immunotherapies in patients with lung cancer underscores the critical need to better understand immunotherapy responses (23). Most genetically engineered models do not fully recapitulate the neoantigen burden (either clonal or subclonal) and/or tumor immune microenvironment. Thus, it is vital to continue developing preclinical models to interrogate cancer immunology and better predict patient responses (bioRxiv 2025.01.15.633175; (24–26)). Developing and refining orthotopic syngeneic and autochthonous *in vivo* models, together with *ex vivo* platforms such as organoids and tissue slice cultures that incorporate immune cells (27,28), should accelerate our understanding of tumor-immune interaction and responses to immune checkpoint and combination immunotherapies.

Pharmacogenomic analyses can link specific cancer genotypes to drug responses. Progress in high-throughput pharmacogenomic profiling using genetically engineered mouse models (27), tumor organoids (17), and patient-derived xenograft models (29) are beginning to accelerate the identification and validation of clinically relevant genotype-drug interactions. *In vivo* studies also have the potential to uncover novel therapeutic targets that may be missed by cell culture analyses and that are impacted by microenvironmental milieus. Integrating these findings with data from clinical trials should further accelerate precision therapy development for lung cancer.

Several lung cancer subtypes are relatively poorly understood, due in part to the lack of robust model systems. Prioritizing the development of new models of squamous/adenosquamous lung cancer, mesothelioma, and large cell neuroendocrine lung carcinoma could greatly improve our understanding of disease mechanisms and therapy responses. In particular, squamous cell lung cancer is a prevalent and aggressive disease with varied molecular features that lacks molecularly targeted therapies and has arguably attracted less research investment over the last two decades. There are limited *in vivo* models that recapitulate the genetics and histology of squamous cell lung cancer (30). Moreover, better understanding of the evolutionary trajectories of squamous cell lung cancer and optimal modeling of distinct genomic subsets are needed, as different driver gene combinations can result in markedly different disease phenotypes and therapeutic responses. Overall, developing new models of understudied lung cancer subtypes should improve our understanding of disease etiology and provide the tools needed to empower drug discovery that will ultimately improve patient outcomes.

### Understanding the Genome, Epigenome, and Proteome Across Lung Cancer Development and Progression

Our understanding of lung cancer biology has been greatly influenced by large-scale genomic and transcriptomic studies of primary tumors. While genomic studies have yielded valuable insights into mutational drivers and resistance mechanisms, they fail to capture the full molecular complexity of lung cancer. Recent studies have demonstrated that non-mutational mechanisms are pivotal in driving cancer phenotypes (31,32). Despite widespread recognition that epigenomic, metabolic, and proteomic processes are essential to all aspects of biology, cancer research has, until recently, focused predominantly on genomic alterations. Future systematic profiling and functional evaluation of epigenomic and proteomic alterations at scale in both preclinical models and clinical samples could have broad implications for identifying new drug targets, improving patient stratification, and advancing precision treatment.

Our understanding of epigenomic regulators beyond those that are genetically mutated remains limited in lung cancer. Changes in the epigenome, including global changes in DNA methylation, chromatin accessibility, and histone modifications can now be systematically analyzed in preclinical models and clinical samples (33–35). Future epigenomic profiling, in combination with functional genomic approaches, could be uniquely valuable for uncovering epigenomic alterations that drive lung tumor growth and shape cellular responses to evolutionary pressures such as therapy and changes in the microenvironment. Moreover, deeper insights into epigenomic drivers at different stages of lung carcinogenesis could inform early detection and expand the repertoire of targets for precision medicine.

Proteomic alterations, encompassing changes in protein expression, post-translational modifications, and protein-protein interactions, often occur independently of genomic alterations (36). These changes can critically impact tumor behavior, drug response, and immune evasion, yet they remain relatively understudied. Ongoing technical advances in high-throughput mass spectrometry (37) and spatial proteomics (38) are beginning to enable increasingly sensitive protein-level analyses that will lead to new biological discoveries and clinical insights. In particular, plasma proteomic analysis leveraging large patient cohorts with clinical metadata, such as the UK Biobank, is promising for improving our understanding of lung cancer risks and prioritizing patients for low-dose CT screening (39).

The genomic understanding of lung cancer, despite many advances, remains incomplete. Even within the receptor tyrosine kinase (RTK)–RAS–MAPK pathway, which is central to many current lung cancer targeted therapies, some genomic alterations are poorly detected by current methods (40). For example, advances in long-read sequencing methodologies provide new opportunities to resolve previously unidentified structural variants (41) and enable more complex transcriptome analysis (42). Systematic long-read sequencing analyses of lung cancer offer great promise to further elucidate the pathogenesis of this genomically driven disease.

Finally, inter- and intra-tumoral heterogeneity, as well as differences between primary tumors and metastases, adds substantial complexity to understanding epigenomic, proteomic, and genomic drivers of lung cancer (27). While certain genomic alterations are enriched in metastases (43), whether they are critical drivers of metastasis and their functional effect on this process remains mostly unclear. Moreover, the epigenomic and proteomic differences between primary tumors and metastases, and how these differences influence therapeutic response and resistance, remain poorly defined. Future large-scale molecular studies of non-mutational heterogeneity using spatial and single-cell multi-omics profiling will be essential to address these gaps. Warm autopsy programs, which enable the collection of multiple metastatic tumor samples from a single patient, offer a unique opportunity to generate comprehensive, multimodal datasets that deepen our understanding of the metastatic process. Such efforts could enable reverse translation to advance our understanding of disease biology, inform biomarkers of cancer progression, and identify new therapeutic strategies.

## Understanding and Overcoming Therapy Resistance

Therapy resistance remains a major barrier to curing lung cancer. Most patients with advanced disease either have intrinsic or eventually develop acquired drug-resistant tumors, reflecting the extraordinary adaptive capacity of tumors and their ecosystems under therapeutic pressure. Resistance mechanisms are complex, often with multiple mechanisms coexisting within the same tumor, shaped by genetic diversification, non-genetic plasticity, lineage transitions, and dynamic interactions among cancer cells, their microenvironment, and the host. It remains unclear whether successive generations of targeted therapies will ultimately exhaust on-target resistance mechanisms or instead drive tumors toward bypass signaling, oncogene amplification, and drug-tolerant persister states. Understanding how these processes unfold over time and across treatment exposures—how tumors adapt, persist, enter dormant states, and acquire resistance—will be key to designing strategies that achieve durable control or eradication.

**Figure (2):**
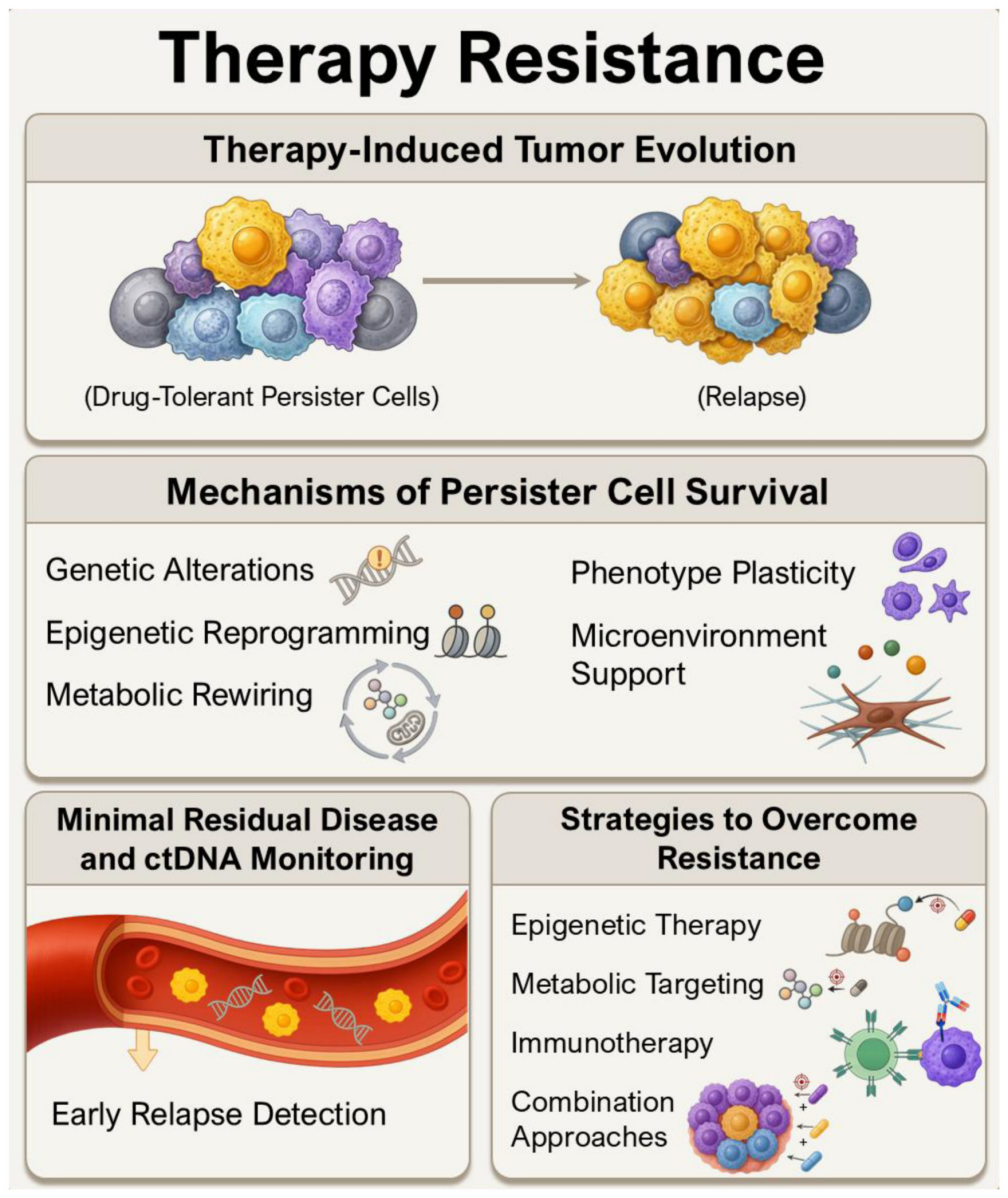
Mechanisms and therapeutic strategies of therapy-induced tumor resistance. Therapy induces the emergence of DTPCs, which can survive initial treatment and subsequently expand to drive tumor relapse. These persister cells utilize multiple survival mechanisms, including genetic mutations, epigenetic reprogramming, metabolic rewiring, phenotypic plasticity, and support from the tumor microenvironment. MRD and ctDNA monitoring enable early detection of relapse by identifying residual disease. Insights into persister cell biology and MRD dynamics can help guide the development of resistance-overcoming therapies, such as epigenetic interventions, metabolic targeting, immune checkpoint blockade, and rational combination approaches.

Investigating the biological foundations of resistance—spanning genetic and epigenetic evolution, the emergence of drug-tolerant persister states, and the role of the tumor microenvironment in shaping therapeutic outcomes—is a key area for future work. A better understanding of how oncogene-addicted tumors with high mutational burden evade the immune system is essential for developing novel therapies that can overcome resistance. (Fig. 2). At the same time, tumor-informed circulating tumor DNA (ctDNA) approaches can detect minimal residual disease and support a shift away from static treatment paradigms toward adaptive, biomarker-guided care in the adjuvant setting. Such strategies may enable treatment escalation in patients with persistent molecular residual disease while allowing de-escalation in those achieving deep molecular responses. By enriching for patients at the highest risk of relapse in drug escalation trials, these approaches could improve cure rates and quality of life while accelerating drug development timelines in the adjuvant setting. Longitudinal molecular profiling, integrated into pragmatic, patient-centric clinical trial designs, will be essential for guiding the next generation of therapeutic interventions. By elucidating the principles governing tumor evolution under therapy and linking these insights to adaptive treatment strategies, resistance may be anticipated and transient vulnerabilities exploited to achieve durable remission and potential cures.

### Towards a Better Understanding of Mechanisms of Tumor Evolution Through Therapy

Lung cancers are dynamic and evolve rapidly in response to therapy (44). An area of unmet need is understanding how different treatments, including targeted therapy, chemotherapy, radiotherapy, and immunotherapy, influence tumor biology and give rise to conserved and treatment-specific resistance mechanisms. Treatment resistance is inevitable in many cases, but the precise molecular and cellular changes that facilitate this adaptation are incompletely characterized. Some critical questions include how the biological properties of tumors shift under therapy, whether resistance to one treatment modality confers vulnerability to another, and the extent to which different therapeutic approaches induce distinct evolutionary trajectories in tumors.

Intrinsic and acquired drug resistance mechanisms in lung cancer are multifaceted. While acquired genetic mutations in key oncogenic drivers such as EGFR, ALK, and KRAS are well documented (45), non-genetic resistance mechanisms remain relatively poorly understood. Cancer cells exhibit remarkable adaptability, shifting within different epithelial states and between epithelial and mesenchymal states, altering their metabolic dependencies, and modifying their immune evasion strategies in response to treatment, all of which can occur independently of additional genetic alterations (46–48). Defining the molecular circuitry that enables this plasticity is central to understanding the mechanisms of resistance. Indeed, it is now well-established that lung adenocarcinomas driven by oncogenic EGFR or KRAS can undergo histological transformation to small cell lung cancer (SCLC; in the case of EGFR mutant tumors) or lung squamous cell carcinoma (for both EGFR and KRAS-driven tumors) upon treatment with targeted therapies (49–51). Investigating how these processes unfold at the single-cell level using high-resolution sequencing and imaging techniques is vital for identifying new points of therapeutic intervention.

Cancer cell state changes likely create novel vulnerabilities that could be exploited to enhance responses to existing treatments, including immunotherapies. For example, KRAS inhibitors have been reported to enhance cancer cell antigen processing, antigen presentation to T cells, and reprogram tumor-associated myeloid cells to assist cancer-specific T cells to traffic and function within tumors (52). These tumor microenvironment changes have the potential to reverse resistance to immune checkpoint agents, improving their activity in patients with non-responsive lung cancers. A better understanding of resistance to KRAS inhibitors and refining predictive biomarkers for patient selection, and rational drug combinations that exploit novel co-dependencies that are not limited to immune checkpoint inhibitors will further advance the field. Moreover, expanding patient benefit across a wider spectrum of KRAS-driven cancers will be fueled by emerging classes of KRAS drugs with greater potency, broader KRAS mutant coverage, and distinct modes of action, such as by targeting the active GTP-bound (ON) state of KRAS (53,54). There is potential for combination targeting with mutant selective and pan RAS inhibitors for optimal suppression of the RAS pathway and prevention of resistance mechanisms (55,56).

### Understanding of the Biology of Drug-Tolerant Disease

Some lung cancer therapies lead to profound tumor responses. However, the persistence of minimal residual disease (MRD) – i.e., microscopic or clinically occult cancer cell populations after systemic therapy, even when radiographic responses indicate maximal tumor shrinkage – can be driven by drug-tolerant persister cells (DTPC) (Fig. 2). Thus, there is a need to develop novel therapeutic strategies that specifically target these resilient cell populations (57). Epigenomic reprogramming likely plays a central role in the development of drug tolerance in lung cancer and targeting this might represent a promising approach. For instance, epigenomic modulators can re-sensitize residual cancer cells to targeted therapies in preclinical models of EGFR-mutant lung cancer (58,59). HDAC inhibitors have also been shown to reprogram myeloid derived suppressor cell populations in mouse and human studies of breast and pancreatic cancers, further enhancing therapeutic outcomes with immune checkpoint blockade (60–63).

DTPCs can undergo metabolic reprogramming towards an increased reliance on oxidative phosphorylation and altered lipid metabolism, which creates unique metabolic dependencies (58,59,64). Enhanced mitochondrial dependency, evidenced by elevated mitochondrial respiration, may render DTPCs susceptible to inhibitors targeting the electron transport chain (65). Furthermore, inhibiting fatty acid oxidation (FAO) has been shown to disrupt persister cell fitness and delay resistance and disease recurrence, highlighting FAO as a therapeutic vulnerability (66). Metabolic inhibitors, either alone or in combination with targeted treatments, could thus provide an effective strategy to reduce DTPCs and delay or prevent disease relapse. Additional levels of plasticity induced by drug treatment can also lead to phenotypic shifts in cancer cells. KRAS-driven lung adenocarcinoma treated with KRAS inhibitors initially exhibit features of alveolar type 2 (AT2) cells but upon treatment withstand therapy by acquiring features of alveolar type 1 (AT1) cells (67). Future pre-clinical research may better define tumor metabolism in DTPCs, and clinical trials should evaluate combination regimens that integrate metabolic agents with targeted therapies to improve treatment durability (Fig. 2).

Existing *in vitro* cell lines and *in vivo* murine models recapitulate aspects, but not all, of the heterogeneity and complexity of drug tolerant resistant disease (DTRD) and a comprehensive understanding of this state from analysis of patient specimens is limited. Clinical protocols rarely include obtaining tissue biopsies at the point of maximal response, restricting access to specimens that include the DTRD state. Most biopsies are performed at progression, missing the opportunity to study DTPCs during treatment-induced remission. Prospective trials incorporating scheduled biopsies at predefined treatment intervals might permit further study of the biology of DTRD. Prospective studies on patient biopsies using emerging multimodal single-cell and spatial technologies to investigate epigenomic, metabolic and microenvironmental changes should be prioritized to better understand DTPC biology.

Targeted therapies can reprogram cancer and inflammatory cells within the tumor immune microenvironment to facilitate the clearance of DTPC (68). Therapeutic strategies that combine targeted agents capable of remodeling the tumor microenvironment to enhance antigen presentation with immune checkpoint inhibitors, are expected to potentiate clinical efficacy beyond either approach alone (Fig. 2). In fact, preclinical data indicate that combining targeted therapies with immune modulators can synergistically promote the clearance of drug-tolerant cells (69). Ongoing clinical trials are evaluating these promising combinations to overcome resistance in lung cancer.

As therapeutic strategies initially developed for advanced disease are increasingly used in neoadjuvant and adjuvant settings, in scenarios analogous to breast cancer, disseminated tumor cells (DTCs) may lie dormant for years or decades after treatment. Studies of the biology of these DTCs, including similarities and differences with DTPCs, will be important, as will understanding the mechanisms that underlie DTC re-awakening (70,71).

### Minimal Residual Disease Assessment for Adjuvant Trial Stratification

Circulating tumor DNA (ctDNA) can serve as a window into minimal residual disease (MRD). The study of ctDNA represents an area of significant promise in advancing a biomarker-driven understanding of lung cancer and transforming patient care (72). Tumor-agnostic and first-generation tumor-informed approaches can stratify early-stage disease both prior to and following surgical resection with curative intent (7,73). Recent work has demonstrated the utility of novel second-generation, ultra-sensitive tumor-informed approaches (74,75). These assays, with the ability to detect ctDNA at plasma concentrations as low as 1–5 parts per million of DNA, can identify ctDNA in the vast majority of patients with NSCLC, including up to 80% of those with adenocarcinomas (74). Detection of ctDNA, even at these low concentrations, is clinically significant, with even low ctDNA levels (e.g., below 80 ppm) being associated with worse outcomes than no ctDNA detection (75). This supports the use of these approaches as part of a ‘TNMB’ (tumor-node-metastasis-blood) staging approach at diagnosis.

Ultrasensitive approaches also promise to enable the dynamic evaluation of the impact of adjuvant therapy, permitting innovative clinical trial approaches wherein patients are selected for adjuvant therapy based on ctDNA detection or kinetics earlier in the disease course in the adjuvant setting (74). The most sensitive assays also provide significant lead times that may allow clinicians to intervene earlier in the context of occult metastatic disease than would be otherwise possible with conventional surveillance (7,74,75). Quantitative changes in ctDNA levels correlate with residual disease burden and treatment response and therefore could serve as a surrogate endpoint in clinical trials (76). Serial ctDNA assessments also permit the tracking of tumor evolution and the early detection of resistance-associated mutations. The field of tumor-informed MRD assessment is primed to optimize adjuvant therapy stratification, allowing the development of new approaches to prevent or intervene when the clonal burden of disease is at a minimum (77).

### Integrating Tumor Profiling and Translational Research

Another pressing challenge in lung cancer treatment is elucidating the evolution of therapy resistance in the presence of macroscopic disease, as opposed to MRD. Approaches using longitudinal, multi-omic profiling of patient samples to investigate the reciprocal interactions between intrinsic tumor molecular changes and the host will be critical to achieving a holistic view of disease progression and treatment response. As discussed above, tumor-based and liquid-based profiling are emerging as tools for decoding lung cancer biology over time (78). The integration of genomic, epigenomic, transcriptomic, and proteomic data from solid and liquid biopsies across the disease course will help us better understand the dynamic mechanisms lung tumors employ to adapt to therapeutic pressure. In addition to studying patient samples, developing *in vivo* models that recapitulate resistant macroscopic lesions would enable molecular characterization and functional perturbation in controlled systems to identify adaptive mechanisms of lung tumors, potentially refining treatment strategies.

High-throughput single-cell sequencing, spatial transcriptomics, and multiplex imaging technologies have the potential to revolutionize our understanding of how tumors respond to therapy at the cellular level. These tools can reveal heterogeneity within tumors, map resistance pathways, and identify novel targets for intervention (79). Further efforts are needed to incorporate these technologies more broadly into clinical research and to ensure that patient-derived data are broadly accessible to enable refinement of preclinical models and inform therapeutic development.

## Opportunities For Novel Therapy Development Across All Lung Cancer Subtypes

**Figure (3):**
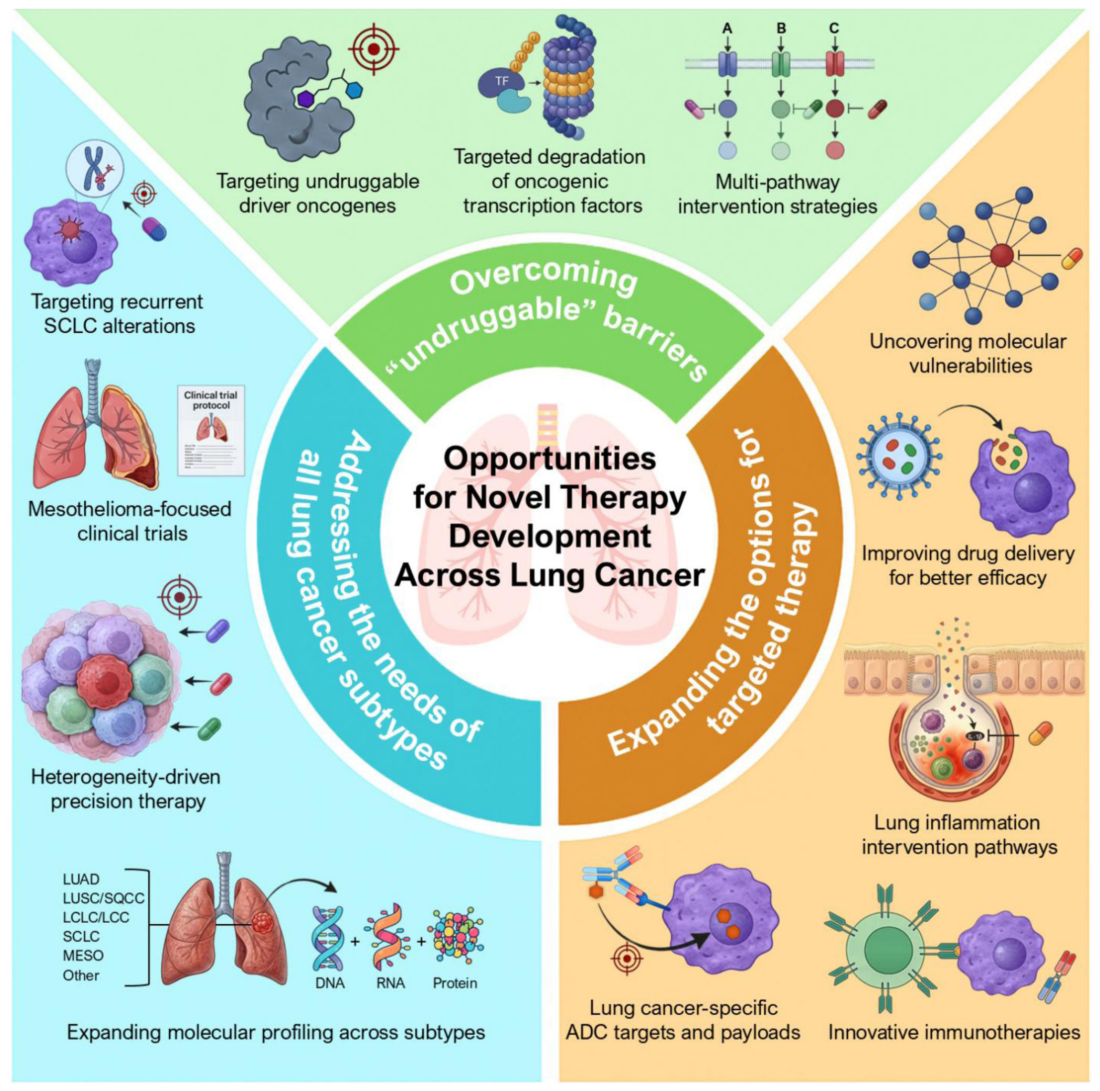
Opportunities for novel therapy development across lung cancer. Key opportunities to extend precision oncology beyond oncogene-defined tumors and to foster durable therapeutic benefit across the full spectrum of lung cancer fall into three interconnected areas: (i) overcoming “undruggable” barriers by exploiting emerging chemistries, such as heterobifunctional protein degraders (proteolysis-targeting chimeras, PROTAC) and molecular glues, to eliminate oncogenic drivers and transcription factors previously considered intractable. This includes pursuing reactivation of mutant tumor-suppressors (e.g., p53), inhibition of oncogenes (e.g., c-MYC), and rational combination strategies targeting compensatory signaling networks; (ii) expanding targeted therapy options by investigating mechanisms in oncogene-negative lung cancers, refining drug delivery approaches such as ADCs to enhance the therapeutic index, and identifying novel surface receptors and payloads unique to lung tumors. Intervention in lung inflammatory pathways also represents a promising therapeutic approach. New molecular dependencies and precision targets may be uncovered by integrating innovative immunotherapies, including CAR T cells and T-cell engagers, with systematic functional genomic screening in preclinical models; (iii) addressing the needs of all lung cancer subtypes by increasing molecular profiling and developing subtype-specific therapeutic strategies. Additionally, mesothelioma-specific clinical trials and reverse translational studies are needed to better understand heterogeneity, plasticity, and other vulnerabilities and evaluate emerging therapeutic agents. (LUAD: lung adenocarcinoma; LUSC/SQCC: lung squamous cell carcinoma; LCLC/LCC: large-cell lung carcinoma; SCLC: small cell lung cancer; MESO: mesothelioma)

Advances in precision medicine, targeted therapies, and immunotherapy have revolutionized cancer treatment, particularly for lung adenocarcinoma, where actionable oncogenic mutations guide treatment. However, a significant proportion of lung adenocarcinomas either do not have known oncogenic drivers or have drivers that are not currently actionable, limiting the breadth of treatment options. For patients with squamous cell lung cancer, SCLC, and other rare subtypes such as mesothelioma and large-cell neuroendocrine carcinoma, there are few effective targeted therapeutic options. Addressing these challenges will require innovative drug discovery approaches, synthetic lethal targeting strategies, and a deeper understanding of drug resistance mechanisms (Fig. 3).

### The Need for Broader Targeted Therapy Options

While approximately half of lung adenocarcinomas harbor actionable oncogenic mutations, with targeted therapies available for EGFR (exon 19 deletions, exon 20 insertion, and L858R point mutations), KRAS-G12C, BRAF, MET, HER2, ALK, ROS1, RET, and NTRK alterations (80), the remaining half and nearly all squamous cell lung cancers lack identifiable oncogenic driver alterations, leaving them mostly without precision-targeted treatment options. New therapies are addressing mutant oncoproteins not previously drugged, including other KRAS variants and KEAP1 mutants (81–87). Investigations to uncover driver mechanisms for oncogene-negative lung cancers are needed to identify novel targets. For example, synthetic lethal approaches to target SMARCA4-deficient lung tumors with SMARCA2 targeting agents and to treat lung tumors with *MTAP* loss with MTA-cooperative PRMT5 inhibitors are under clinical investigation (88,89).

Developing more effective drug delivery approaches could improve therapeutic efficacy and quality of life outcomes. Antibody-drug conjugates (ADCs), which combine the potent cytotoxic effect of the conjugate payload with the targeting specificity of monoclonal antibodies to cell surface antigens, have demonstrated promise in patients. ADCs targeting TROP2, HER2, HER3, c-MET, B7H3, and CEACAM5 are either approved or under investigation for the treatment of NSCLC, and similar agents against other targets are showing promise in SCLC patients (90). Identifying novel and stable lung cancer cell surface targets (ideally expressed on most cancer cells) and exploring innovative payloads and antibody formats could significantly broaden the scope of ADC therapies (91). While challenges such as antigen variability and adverse effects remain, advances in ADC design, safety protocols, and combination therapies to combat resistance will be essential as these agents move forward. Identifying additional cell surface proteins unique to NSCLC, SCLC, and other subtypes will be critical in developing next-generation ADCs that selectively deliver cytotoxic agents to cancer cells while sparing normal tissues. Ultimately, ADC development will need to balance efficacy and safety. Efficacy may be driven by molecular heterogeneity and conjugation, leading to variable drug-to-antibody ratios, which in turn result in unpredictable pharmacokinetics and compromised efficacy. Linker instability and premature payload detachment into the circulation can lead to off-target toxicity, but these issues may be mitigated by advances in linker technologies, such as lysosomal protease–cleavable linkers. There has also been a surge in the testing of different payloads, including novel and dual-payload ADCs, to address the major challenge of cross-resistance across ADCs due to the use of similar payloads.

Bispecific T-cell engagers represent another exciting class of emerging immunotherapy strategies with applications for the targeted eradication of lung cancer cells. Tarlatamab, which binds CD3 on T cells and DLL3 on cancer cells, demonstrated superiority over chemotherapy (hazard ratio for survival 0.60) for the treatment of patients with recurrent metastatic SCLC and was approved by the US FDA (92). Other similarly designed T-cell engagers have also shown activity in early phase studies in patients with SCLC and other high grade neuroendocrine cancers (93). The exceptional clinical activity of T-cell engagers in patients with SCLC versus other solid tumors might relate to the frequent suppression of antigen processing and presentation machinery in SCLC – a mechanism of immune escape associated with resistance to PD-(L)1-directed immunotherapy but bypassed by T-cell engagers (94).

Several additional biological features of SCLC may also contribute to the relative success of T-cell engagers in this disease and may shed light on strategies for NSCLCs that are less responsive. First, SCLC typically exhibits high and relatively homogeneous expression of lineage-specific surface antigens such as DLL3, providing a dense and uniform target for T-cell redirection, whereas NSCLC is characterized by greater antigen heterogeneity and lower expression of most targetable surface proteins. Second, SCLC tumors often have less stromal fibrosis and physical barriers to immune cell infiltration compared with many NSCLCs, potentially allowing more efficient T-cell–tumor cell contact once T cells are redirected by the engager. Third, the neuroendocrine phenotype of SCLC may be associated with reduced intrinsic immune signaling and antigen presentation, making these tumors poorly responsive to checkpoint blockade but uniquely susceptible to therapies that forcibly recruit and activate T cells independent of major histocompatibility complex presentation. Understanding these disease-specific biological contexts will be critical for identifying optimal targets, combination strategies, and patient populations for next-generation T-cell engager approaches across all lung cancer subtypes.

The future of immunotherapy in lung cancer will likely involve moving promising agents into earlier therapeutic settings and stages of disease and the use of rational combinations. Ongoing research should help overcome resistance to immune checkpoint inhibitors and improve patient selection strategies through the development of novel predictive biomarkers of response and resistance (95).

Finally, systematic identification of driver genes in clinically relevant model systems, expanding screens for additional molecular vulnerabilities, and the incorporation of additional innovative therapeutic strategies, including CAR T cells, and neoantigen vaccines are needed to narrow the gaps for lung cancer patients lacking actionable precision oncology approaches.

### Overcoming the “Undruggable” Barrier in Lung Cancer Therapy

A major limitation of current lung cancer treatment is the difficulty in targeting known oncogenic drivers, either due to the lack of well-defined binding pockets or their complex interactions within pathways. It is estimated that current therapeutic strategies exploit less than 10% of known cancer-driving proteins (96), underscoring the vast untapped potential in lung cancer drug development. Advances in chemistry, target biology and data science are revolutionizing cancer drug discovery, leading to the identification of new targets and drug designs against previously undruggable targets (97). One key advance has been targeted protein degradation which reprograms the target specificity of the ubiquitin-proteasome system to induce selective ubiquitination and degradation of oncogenic drivers. Rapid progress has been made in the design of heterobifunctional proteolysis targeting chimeras (PROTACs) and molecular glue degraders, targeting key lung cancer culprits, such as KRAS (84,98,99). These novel therapeutic strategies could tackle traditionally challenging targets, including oncogenic transcription factors. Protein degraders and molecular glues also allow for higher treatment specificity, deeper and more sustained duration of drug effects and potentially limited therapeutic resistance, while minimizing toxicity, ultimately providing the potential for greater patient benefit. Drugging ‘undruggable’ targets will continue to be an important research priority, with expanded approaches to reactivate mutant p53 or otherwise therapeutically exploit p53 mutations, and efforts to inhibit currently intractable oncogenic drivers.

Beyond inhibiting single targets, combination therapies using a diversity of modalities will likely be required. As signaling pathways are rife with redundancy and feedback loops, inhibition of a single pathway often leads to compensatory mechanisms that drive resistance (100,101). Systems biology approaches will contribute to map complex cancer networks and identify optimal nodes for intervention. Simultaneously targeting multiple pathways should reduce the likelihood of cancer cells developing resistance, resulting in more durable drug responses.

### Addressing the Needs of All Lung Cancer Subtypes

Although substantial therapeutic advances have been made for lung adenocarcinoma, options for other lung cancer subtypes, which account for over 50% of patients, remain limited. SCLC lacks effective targeted therapies primarily due to the absence of actionable genomic alterations (102) and there is a shortage of biomarker-guided clinical trials for these patients. The most frequent genetic events in SCLC are the inactivation of *RB1* and *TP53*, as well as *MYC* family amplifications, all of which present significant drug development challenges. However, recent advances in antibody-based therapies, such as ADCs and T cell engagers, have emerged as promising options. As mentioned above, the approval of the DLL3xCD3 bispecific T cell engager Tarlatamab represents a breakthrough in SCLC treatment (92,103) and could spur new developments for antibody-based therapies (104). Additionally, synthetic lethal strategies, mutant p53 correctors, and Aurora A inhibitors targeting MYC-dependent cancers are making headway as potential therapies for SCLC (105,106). Continued efforts to understand the heterogeneity and phenotypic plasticity of SCLC subtypes (107,108), and uncovering non-mutated vulnerabilities will also be crucial for developing more effective precision treatments.

Mesothelioma is another particularly challenging malignancy due to its aggressive nature and limited treatment options. Recent studies on mesothelioma genomics and underlying biology have laid the groundwork for potential targeted therapies. For instance, YAP/TEAD inhibitors have shown preliminary antitumor activity. One such inhibitor has received FDA orphan drug and fast track designations for the treatment of patients with mesothelioma, providing a foundation for further exploration (109). However, the transition from early signs of efficacy to clinically meaningful treatments has been hindered by a relative paucity of dedicated research funding and clinical trial infrastructure for mesothelioma. Establishing mesothelioma-specific clinical trials that evaluate emerging therapeutics, including targeted inhibitors and immunotherapy combinations, should be prioritized. Reverse translational studies should further elucidate the underlying biology of mesothelioma and identify new targets for intervention. Expanding *in vivo* and *ex vivo* model systems, increasing research funding, and fostering clinical trials based on a deeper understanding of mesothelioma biology will be instrumental for building a pipeline to translate preclinical discoveries to clinical applications.

Similarly, other lung cancer subtypes, such as large-cell neuroendocrine carcinoma and adenosquamous carcinoma, lack targeted therapeutic strategies. These lung cancer subtypes remain poorly characterized biologically (110,111); thus, expanding molecular profiling efforts and integrating these data into precision oncology trials will be necessary to identify targetable alterations and refine treatment approaches.

## Clinical Trial Advances

Advances in lung cancer therapeutics have arguably been driven as much by innovation in clinical trial design as by breakthroughs in biology. The next phase of progress will depend on reimagining how clinical trials are conceived, conducted, and integrated with real-world data to accelerate discovery and broaden patient benefit. Lung cancer trials increasingly intersect with large-scale genomic, phenotypic, and digital datasets, thus enabling a more precise understanding of therapeutic response, resistance, and toxicity. However, the field remains hampered by fragmented data, inconsistent genotyping and phenotyping standards, and uneven representation of key demographic and ethnic groups. Emerging priorities to overcome these challenges include harmonizing molecular and clinical profiling across studies, ensuring inclusivity and diversity in trial populations, redefining clinical endpoints to reflect both efficacy and quality of life, and building centralized, artificial intelligence (AI)-enabled repositories to enhance data sharing and accessibility. Together, these advances will enable more agile, globally connected, and biologically informed clinical research, ensuring that each patient’s experience contributes to the collective acceleration of lung cancer care.

### Consistent Genotyping and Phenotyping

Harmonizing approaches for genotyping and phenotyping in clinical trials and real-world treatment settings could facilitate cross-trial comparisons, lead to novel disease insights, and improve predictive models for lung cancer therapy. Large-scale data-sharing initiatives such as AACR Project GENIE (112) have revolutionized our understanding of cancer mutation frequencies and empowered basic and translational research by making these data readily accessible. However, the impact of these genomic databases would be further amplified by more extensive integration of genomic profiles with other clinical information, such as histopathology, treatment modalities, and clinical outcomes (113). Future integration of clinical trial data with other real-world clinical evidence would provide a comprehensive view of treatment efficacy across diverse patient populations (114). Furthermore, trials investigating drugs with novel mechanisms of action should prioritize generating and sharing clinical data to maximize the value of each patient and advance the field’s understanding of emerging cancer biology. By aligning clinical findings with pre-clinical studies, response patterns and resistance mechanisms may be better understood and predicted, ultimately leading to more precise and effective therapeutic interventions.

A fundamental capability of AI is its ability to integrate and derive insights from large heterogeneous datasets. Organizing and centralizing mutational, clinical, and other molecular data, such as diagnostic imaging, epigenomics, and proteomics, would provide foundational resources for AI-assisted approaches to advance precision oncology. Such resources could support hypothesis generation, biomarker discovery, and the development of more accurate models for predicting treatment response.

### Advancing Lung Cancer Research in Distinct Populations

Lung cancer is a heterogeneous disease at multiple levels with significant disparities in incidence, molecular characteristics, and treatment outcomes across different populations. Addressing these disparities will require age-, sex- and race/ethnicity-specific research, inclusivity within trials, and establishing global lung cancer registries. We envision a future where the more precise incorporation of these key patient demographics and other factors contribute to optimized screening and precision lung cancer treatments.

Age likely impacts multiple aspects of lung cancer biology and treatment response. Young adults with lung cancer (<50 years) represent a distinct and clinically important population. Unlike most lung cancer patients, these patients are often never-smokers, suggesting that other environmental exposures and/or genetic predispositions play a larger role in disease development (115). While this population has a higher prevalence of oncogenic fusion-driven lung cancer (116) which typically confer sensitivity to targeted therapies, long-term disease control remains a challenge. Moreover, lung tumors in young patients generally have a much lower mutation burden than their counterparts in older smokers (117). Future investigations of the biology of these tumors, their routes to resistance, and interactions with the immune environment could uncover important and clinically meaningful differences (118). Conversely, lung tumors in very old patients likely possess unique cell intrinsic and microenvironmental characteristics (119), thus additional consideration of age across pre-clinical and clinical studies will likely be of value.

Lung cancer has been the leading cause of cancer-related deaths among women in the United States since 1987 (120), yet sex-specific differences in disease development, progression, interactions with immune and stromal cells, and therapy responses remain underexplored. The rising prevalence of lung cancer in women, particularly among never-smokers, suggests that hormonal, genetic, and environmental factors may play critical roles in the pathogenesis of this subset of tumors (121). In addition to biological factors, psychosocial and economic issues disproportionately affect female lung cancer survivors. Women often face unique survivorship challenges, including caregiving responsibilities and financial strain (122). These realities underscore the pressing need for increased research on lung cancer in women, greater inclusion of women in clinical trials, and sex-specific analyses of treatment responses in clinical trials and real-world datasets (123,124).

While lung cancer is tightly associated with smoking, the high incidence of lung cancer in never-smokers, particularly among Asian populations is alarming (125). Among Asian American ethnic groups, lung cancer ranks among the top three causes of cancer-related deaths (126) and in Chinese, Vietnamese, Japanese, and Korean populations, it remains the leading cause of cancer mortality (127). The incidence of lung cancer in never-smokers is notably higher among Asian women. For example, 83% of Filipino women and 98% of Asian Indian women diagnosed with lung cancer have never smoked (128). While lung cancer in Asian never-smokers is enriched for some genomic alterations, most notably EGFR mutations, the basis for the observed ethnic and gender disparities in lung cancer spectra in never-smoking Asian individuals remains poorly understood. Genetic predisposition and environmental exposures including air pollution (9) are thought to play a role, yet few studies have systematically examined these risk factors (129,130). The interplay between genetic susceptibility and environmental exposures is an area requiring further investigation (131).

There remains an urgent need to understand the risk factors, screening strategies, and treatment approaches for lung cancer across distinct populations. Addressing these challenges will require dedicated research initiatives, international registries for young and non-smoker lung cancer patients that include race/ethnicity, lifestyle factors, and environmental exposure information.

### Novel Clinical Trial Endpoints

Lung cancer clinical trials have historically focused on progression-free and overall survival as primary endpoints. However, future trial frameworks should expand to include systematic evaluation of dosing regimens and treatment duration, an area now strongly supported by regulators. For example, the FDA’s Project Optimus initiative is reforming dose optimization and selection in oncology drug development, aiming to ensure that drug schedules are rigorously characterized before registrational trials rather than defaulting to the maximum tolerated dose. This shift is especially important as patients may remain on novel therapies for prolonged periods, where the goal is not only to extend survival but also to maintain or improve quality of life. Dose optimization is particularly relevant for consolidation therapy in early-stage disease and maintenance strategies in advanced-stage settings, where the presence or absence of MRD may critically influence treatment stratification and benefit. Prolonged therapy potentially increases cumulative toxicity and imposes substantial financial burden, underscoring the need for adaptive approaches. As mentioned above, incorporating biomarkers such as longitudinal ctDNA analysis into trial designs, could refine patient selection, enable treatment de-escalation or intensification based on MRD status, and ultimately improve both clinical outcomes and sustainability of care. Such sequential molecular testing could enable the detection of emerging resistant clones, which may be used to guide the specific choice of future therapies.

The Lung-MAP protocol is a groundbreaking master protocol clinical trial designed to transform how therapies are tested in advanced NSCLC. Rather than launching separate studies for every targeted therapy, Lung-MAP uses a single, centralized genomic screening platform to analyze a patient’s tumor and then assigns them to a biomarker-matched sub-study or a non-match investigational arm, dramatically reducing screen-fail rates and accelerating the pace of clinical research. This adaptive framework allows new drugs and biomarker-defined cohorts to be added as the science evolves, creating a living, continuously updated trial that reflects the rapidly changing landscape of lung cancer treatment (132). Lung-MAP also serves as a major translational research engine, collecting tumor tissue, blood, and genomic data from thousands of patients, enabling investigators to explore mechanisms of resistance, evaluate emerging biomarkers, and link molecular features to clinical outcomes (133). Its collaborative structure illustrates how coordinated national efforts can accelerate precision medicine in oncology.

Building on this momentum, Project Pragmatica represents the FDA’s initiative working with the lung cancer cooperative groups to modernize oncology trials by integrating pragmatic design elements that make studies faster, less burdensome, and more reflective of real-world clinical practice. The Pragmatica-Lung trial is the flagship example of this radically simplified approach (134). With broad eligibility criteria, minimally required tests, limited data collection, reliance on routine clinical imaging, and streamlined monitoring, Pragmatica-Lung enrolled rapidly across academic and community sites and reached a far more diverse patient population than typical oncology trials. While the investigational combination did not improve overall survival, the trial successfully demonstrated that pragmatic designs could maintain scientific integrity while dramatically reducing complexity, cost, and time to completion. Another example is the adaptive phase 2 umbrella study CTONG1702 investigating pyrotinib, an irreversible tyrosine kinase inhibitor against EGFR, HER2, and HER4, in treatment naive, advanced HER2-mutant NSCLC. This study utilized a patient-centric, modern trial design that integrated real-world cohorts and a compassionate use arm, thereby including patients typically excluded from clinical trials (135). The trial cohort demonstrated an ORR of 35.7% and median PFS of 7.3 months. These findings demonstrated the importance of bridging precision oncology with inclusive methodologies to better serve diverse lung cancer patient populations.

These trials illustrate complementary innovations in clinical trial design, focused on precision-medicine matching or simplifying trial processes. Their learnings and successes should drive further advances in lung cancer clinical trial design.

### Data Sharing and Centralization of Lung Cancer Clinical Trial Data

A key barrier to advancing clinical research in lung cancer is the fragmentation of trial data and the lack of accessible, up-to-date information for clinicians, basic science researchers, and patients. The current clinicaltrials.gov database, while a valuable resource, often suffers from outdated or incomplete listings, making it challenging for patients to identify relevant trials and for clinicians to guide enrollment. To overcome this limitation, a platform should provide continuously updated information on ongoing and upcoming clinical trials, detailing eligibility criteria, biomarker requirements, and investigator or study site contact details. Such a system would facilitate greater trial participation, improve patient access to innovative therapies, and enhance collaboration between research institutions.

Furthermore, integrating AI-driven analytics into the database could help match patients with suitable trials based on their genomic and clinical profiles. Leveraging AI on complex datasets could help offer more personalized trial recommendations, thereby optimizing patient enrollment and ensuring that novel therapies reach the appropriate populations in a timely manner.

## Early Detection, Prevention, and Interception Strategies

Prevention and early detection of lung cancer offer clear opportunities to improve survival, yet most patients are still diagnosed at an advanced stage. While tobacco cessation remains the most effective preventive measure, additional strategies are urgently needed to address the existence of environmental carcinogens, including particulate matter, radon exposure, and occupational hazards. Promising avenues such as chemoprevention and emerging “preventative vaccines” targeting oncogenic drivers like *KRAS* and *EGFR* in high-risk populations, underscore the potential for a more comprehensive approach to disease prevention and interception. Public health initiatives, from stricter air quality regulations to genetic risk assessment and screening, should be prioritized to broaden the reach and impact of these measures. Such efforts would ensure that prevention and early detection are embedded as central pillars in reducing the global burden of lung cancer.

### Early Detection Strategies

While the five-year survival rate for lung cancer remains among the lowest of all cancer types, tumors detected at early stages are associated with significantly better outcomes (136). Several trials have demonstrated a mortality reduction from low-dose computed tomography (LDCT) scans in high-risk populations defined predominantly by age and smoking history (137). Screening programs in different regions have different entry criteria, reflecting differences in epidemiology, health care infrastructure, and the ongoing debate over the optimal balance of benefit and harm at both the individual and population level (138). Screening in the United States has been associated with a shift in the stage distribution with a 4.6% annual decrease in distant-stage disease incidence during 2013-2019, while the rate for localized-stage disease rose by 3.6% annually (139). Although the recommended eligibility criteria for lung cancer screening have been broadened, uptake remains low (140).

With the current algorithms for entry criteria, many smokers at lower risk remain ineligible for screening. Research to better understand the risk in individuals with shorter smoking duration or lower intensity, barriers to screening uptake and adherence, will be important. Blood, nasal, and bronchial biomarkers are also being developed. With further research, these methods could better identify patients most likely to benefit from lung cancer screening and thereby enhance early detection. Notably, the FDA has approved biomarkers for estimation of risk but not for early detection (141). Multi-cancer early detection tests are also being developed, and their role in lung cancer screening will be an important area of investigation.

Lung cancer LDCT screening remains complicated by false-positives and the potential for overtreatment. More sophisticated and integrated approaches will be required for screening to benefit lower risk individuals. While diameter assessment and volumetry can help determine category and guide subsequent evaluation (142), machine learning has also been used to aid radiologists for both detection and diagnosis (143). Biomarkers to categorize indeterminate pulmonary nodules are an active area of investigation, and several have received FDA approval (144). However, invasive biopsy procedures are still needed to confirm malignancy and better define treatment, and these invasive procedures carry risks. Moreover, indolent, low-grade malignancies may be discovered, and aggressive treatment may offer more harm than benefit. Enhancing screening quality through improved imaging protocols, standardized management of nodules, and robust smoking cessation support will be critical to ensure that the benefits of early detection translate into meaningful reductions in lung cancer mortality.

Never smokers and younger individuals at risk lack effective screening and early detection programs. Current lung cancer screening guidelines leave younger patients and never smokers undiagnosed until they present with advanced disease. Additionally, the incomplete understanding of hereditary and environmental risk factors contributes to the challenge of identifying high-risk individuals in this age group. The absolute number and proportion of lung cancer in never smokers is expected to increase (145–147). Some modeling suggests that there may be identifiable groups such as those with family histories or with severe air pollution exposure histories at high enough risk to consider screening. However, measuring individual level exposure precisely enough for inclusion in these risk models is difficult (148), pointing to the unmet need to identify blood or sputum biomarkers that enable risk stratification for future lung cancer diagnosis.

The pattern of lung cancer in never-smokers is different in East Asian countries and single-arm LDCT studies have been conducted to address the higher incidence and mortality in these populations (149,150). The Taiwan Lung Cancer Screening in Never-Smoker Trial (TALENT) enrolled >12,000 people, most of whom were non-smokers, enriched for females, and those with a family history of lung cancer. While a negative chest x-ray was required for entry, baseline LDCT scans identified >300 patients with carcinomas of which almost all were adenocarcinoma, >75% were stage I disease, and many were adenocarcinoma *in situ* (149). The frequency of false positives in this population needs to be better evaluated. Ground-glass opacities are frequently identified and usually are associated with more indolent, early-stage adenocarcinoma or adenocarcinoma *in situ*. Better criteria for determining when to intervene are needed, particularly in elderly patients who are more likely to die from other causes. Finally, reduction in late-stage cancer in the screened arm is often considered to be a sufficient intermediate endpoint as it has been shown to track very closely with overall reduction in lung cancer mortality and can substantially accelerate study timelines in this critical area (151).

### Prevention and Interception Strategies

**Figure (4):**
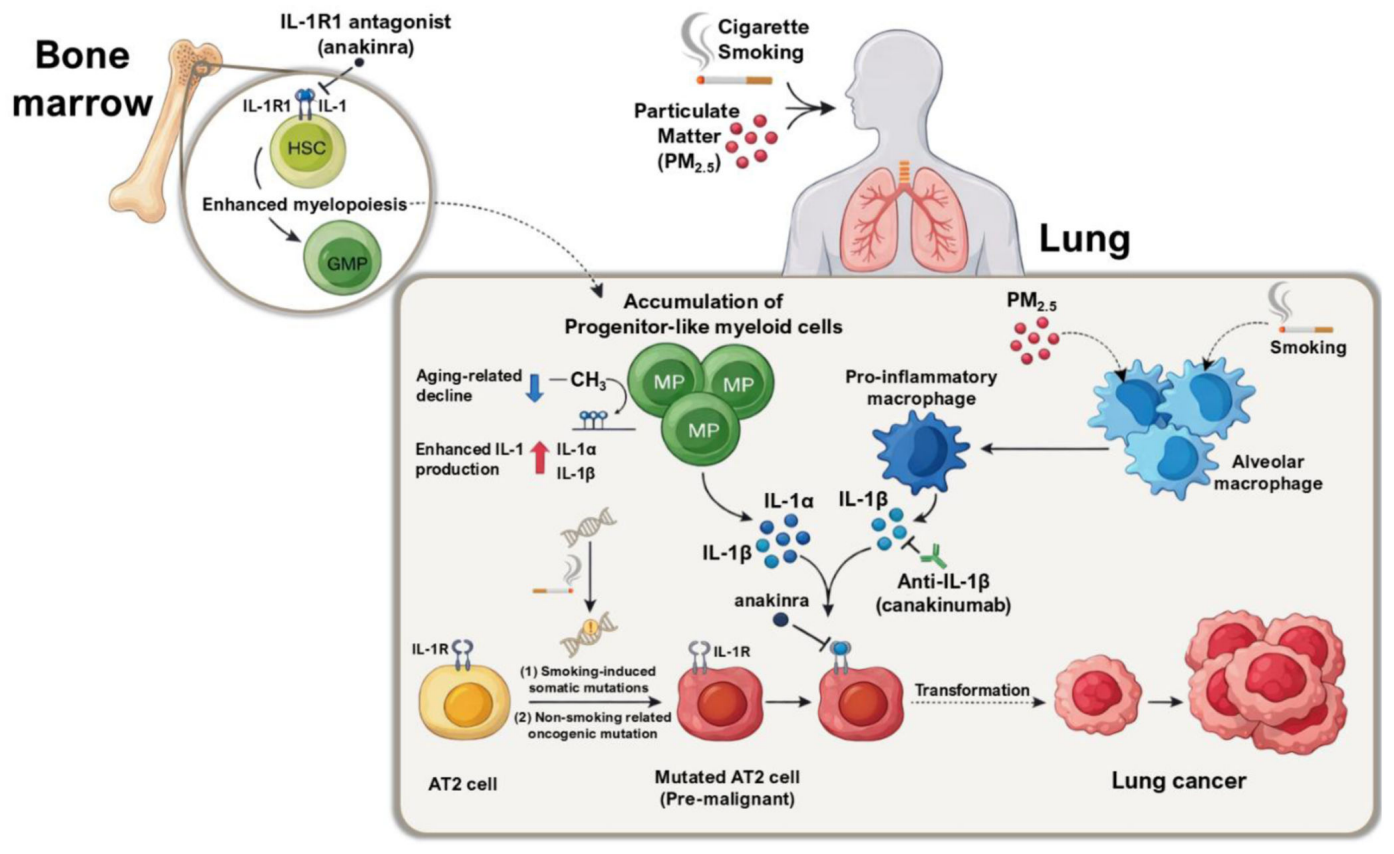
Hypothetical model of IL-1 signaling as a convergence point for CHIP and particulate matter–driven lung tumor promotion, with opportunities for interception. Aging-associated CHIP drives IL-1α/β secretion and enhanced myelopoiesis, whereas cigarette smoke and PM2.5 further amplify pulmonary IL-1β level via proinflammatory macrophage activation. This converging inflammatory microenvironment could act upon premalignant AT2 cells harboring oncogenic driver mutations (e.g., KRAS and EGFR) to promote malignant transformation. IL-1R antagonism (anakinra) and anti-IL-1β therapy (canakinumab) represent tractable interception strategies, providing a mechanistic rationale for chemoprevention trials in high-risk individuals.

While progress has been made over the last decade in identifying high risk patients, there have been no molecular prevention trials designed to reduce lung cancer incidence in high-risk groups. IL-1β is emerging as a critical lung cancer promoter. Released by macrophages in the lung microenvironment in response to particulate matter exposure from the environment or cigarette smoke (9), and from clonal hematopoiesis of indeterminate potential (CHIP) (152), IL-1β links the aging-related process of clonal hematopoiesis with environmental exposures through common inflammatory pathways (Fig. 4). It is increasingly appreciated that cytokines, including IL-1α and IL-1β, are released from tissue infiltrating monocytes derived from CHIP which contribute to lung tumor growth in animal models (153). CHIP is associated with lung cancer incidence in the UK Biobank (152) and CHIP infiltrating primary NSCLC (termed TI-CH or tumor infiltrating clonal hematopoiesis) is associated with increased risk of death or recurrence in NSCLC (8). Mechanistically, IL-1β acts on alveolar type 2 (AT2) cells, a cell-type of origin for lung adenocarcinoma, to enhance their progenitor stem cell capacity that is further exacerbated in the presence of an oncogenic EGFR mutation (9,10) (Fig. 4). Consistent with this model, an analysis of the cardiovascular disease prevention trial CANTOS reported a dose-dependent association between the blockade of IL-1β with reduced lung cancer incidence (154). The failure of IL-1β blockade as an adjuvant or metastatic treatment emphasized a likely unique “hit-and-run” role of IL-1β during the earliest stages of tumor initiation. To date, no other trials are testing IL-1 blocking agents for lung cancer prevention (154,155). IL-1 blockade represents one avenue among many immunomodulatory strategies and other immune or targeted agents that could offer opportunities for preventive interventions.

Preventive vaccines for lung cancer represent a pioneering frontier in oncology. Building on the success of HPV and HBV vaccines, researchers are now developing innovative vaccine approaches targeting oncogenic drivers like *KRAS, EGFR*, and other early oncogenic neoantigens to intercept cancer before it emerges (156). For example, LungVax is a lung cancer prevention vaccine designed to train the immune system to recognize common neoantigens in ever-smokers, aiming at providing protection especially in high-risk individuals (157). The development of robust preclinical models for prevention could aid in assessing the large number of putative contributors to tumor initiation.

The absence of molecular prevention trials is partly attributable to the lack of a robust commercial model for lung cancer prevention. Unlike treatments for established cancers, preventive strategies often do not offer comparable short term financial incentives. Trials of therapeutics aimed at reducing lung cancer incidence have also been hindered by long time horizons for outcome measurement, challenges in establishing appropriate endpoints, and uncertainties regarding overall benefit in otherwise healthy individuals. Importantly, the barrier to approval of such prevention therapies is likely to be higher than for systemic therapies in patients with established disease, given the need to balance benefit against risk and toxicities in otherwise healthy, disease-free individuals. The future development of innovative funding models and collaborative partnerships could stimulate the development of molecular prevention trials and ultimately bridge the gap between risk identification and effective intervention.

## Conclusions and Future Directions

Lung cancer research stands at a pivotal inflection point. Decades of work in genomics, immunology, and clinical investigation have revolutionized our understanding of lung cancer biology and delivered meaningful survival gains for subsets of patients. Advances in basic research, biotechnology, translational research, and data science offer tremendous opportunities to continue transforming patient care through early detection, prevention, and effective precision therapeutic interventions. Addressing critical knowledge gaps to develop a comprehensive understanding of disease mechanisms, overcome resistance to existing therapies, develop new interventions, advance clinical trials, and improve early detection and disease prevention requires concerted, interdisciplinary efforts. The AACR Lung Cancer Task Force is committed to spearheading these efforts, advocating for innovative funding models, and fostering collaborative research partnerships. Through these key initiatives, we aim not only to reduce lung cancer mortality, but also to achieve long-term disease control and move closer to a cure.

## Statement of Significance

Despite substantial progress in understanding disease biology and improving therapy, lung cancer remains the leading cause of global cancer-related deaths. The members of the AACR Lung Cancer Task Force describe areas of unmet need and define near-term research priorities and opportunities to reduce lung cancer morbidity and mortality.

## References

[1] Chhikara BS, Parang K (2023). Global Cancer Statistics 2022: the trends projection analysis. Chemical Biology Letters.

[2] Al Bakir M, Huebner A, Martínez-Ruiz C, Grigoriadis K, Watkins TBK, Pich O (2023). The evolution of non-small cell lung cancer metastases in TRACERx. Nature.

[3] Frankell AM, Dietzen M, Al Bakir M, Lim EL, Karasaki T, Ward S (2023). The evolution of lung cancer and impact of subclonal selection in TRACERx. Nature.

[4] Rosenthal R, Cadieux EL, Salgado R, Al Bakir M, Moore DA, Hiley CT (2019). Neoantigen-directed immune escape in lung cancer evolution. Nature.

[5] McGranahan N, Rosenthal R, Hiley CT, Rowan AJ, Watkins TBK, Wilson GA (2017). Allele-Specific HLA Loss and Immune Escape in Lung Cancer Evolution. Cell.

[6] Abbosh C, Birkbak NJ, Wilson GA, Jamal-Hanjani M, Constantin T, Salari R (2017). Phylogenetic ctDNA analysis depicts early-stage lung cancer evolution. Nature.

[7] Abbosh C, Frankell AM, Harrison T, Kisistok J, Garnett A, Johnson L (2023). Tracking early lung cancer metastatic dissemination in TRACERx using ctDNA. Nature.

[8] Pich O, Bernard E, Zagorulya M, Rowan A, Pospori C, Slama R (2025). Tumor-Infiltrating Clonal Hematopoiesis. New England Journal of Medicine.

[9] Hill W, Lim EL, Weeden CE, Lee C, Augustine M, Chen K (2023). Lung adenocarcinoma promotion by air pollutants. Nature.

[10] Choi J, Park J-E, Tsagkogeorga G, Yanagita M, Koo B-K, Han N (2020). Inflammatory Signals Induce AT2 Cell-Derived Damage-Associated Transient Progenitors that Mediate Alveolar Regeneration. Cell Stem Cell.

[11] Alonso-Curbelo D, Ho Y-J, Burdziak C, Maag JLV, Morris JP, Chandwani R (2021). A gene-environment-induced epigenetic program initiates tumorigenesis. Nature.

[12] Enfield KSS, Colliver E, Lee C, Magness A, Moore DA, Sivakumar M (2024). Spatial Architecture of Myeloid and T Cells Orchestrates Immune Evasion and Clinical Outcome in Lung Cancer. Cancer Discov.

[13] Al-Sawaf O, Weiss J, Skrzypski M, Lam JM, Karasaki T, Zambrana F (2023). Body composition and lung cancer-associated cachexia in TRACERx. Nat Med.

[14] Politi K, Zakowski MF, Fan P-D, Schonfeld EA, Pao W, Varmus HE (2006). Lung adenocarcinomas induced in mice by mutant EGF receptors found in human lung cancers respond to a tyrosine kinase inhibitor or to down-regulation of the receptors. Genes Dev.

[15] Meuwissen R, Linn SC, Linnoila RI, Zevenhoven J, Mooi WJ, Berns A (2003). Induction of small cell lung cancer by somatic inactivation of both Trp53 and Rb1 in a conditional mouse model. Cancer Cell.

[16] Jackson EL, Willis N, Mercer K, Bronson RT, Crowley D, Montoya R (2001). Analysis of lung tumor initiation and progression using conditional expression of oncogenic K-ras. Genes Dev.

[17] Kim M, Mun H, Sung CO, Cho EJ, Jeon H-J, Chun S-M (2019). Patient-derived lung cancer organoids as in vitro cancer models for therapeutic screening. Nat Commun.

[18] Sánchez-Rivera FJ, Papagiannakopoulos T, Romero R, Tammela T, Bauer MR, Bhutkar A (2014). Rapid modelling of cooperating genetic events in cancer through somatic genome editing. Nature.

[19] Cai L, Wu F, Zhou Q, Gao Y, Yao B, DeBerardinis RJ (2025). The Lung Cancer Autochthonous Model Gene Expression Database Enables Cross-Study Comparisons of the Transcriptomic Landscapes Across Mouse Models. Cancer Res.

[20] Hebert JD, Xu H, Tang YJ, Ruiz PA, Detrick CR, Wang J (2025). Efficient and multiplexed somatic genome editing with Cas12a mice. Nat Biomed Eng.

[21] Tang YJ, Shuldiner EG, Karmakar S, Winslow MM (2023). High-Throughput Identification, Modeling, and Analysis of Cancer Driver Genes In Vivo. Cold Spring Harb Perspect Med.

[22] Hebert JD, Neal JW, Winslow MM (2023). Dissecting metastasis using preclinical models and methods. Nat Rev Cancer.

[23] Skoulidis F, Araujo HA, Do MT, Qian Y, Sun X, Cobo AG (2024). CTLA4 blockade abrogates KEAP1/STK11-related resistance to PD-(L)1 inhibitors. Nature.

[24] Cui C, Wang J, Fagerberg E, Chen P-M, Connolly KA, Damo M (2021). Neoantigen-driven B cell and CD4 T follicular helper cell collaboration promotes anti-tumor CD8 T cell responses. Cell.

[25] DuPage M, Cheung AF, Mazumdar C, Winslow MM, Bronson R, Schmidt LM (2011). Endogenous T cell responses to antigens expressed in lung adenocarcinomas delay malignant tumor progression. Cancer Cell.

[26] Exposito F, Connolly KA, Tang T, Chiorazzi M, Hunt BG, Cardenas JJ (2025). Preclinical Models of Solid Cancers for Testing Cancer Immunotherapies. Annu Rev Cancer Biol.

[27] Li C, Lin W-Y, Rizvi H, Cai H, McFarland CD, Rogers ZN (2021). Quantitative In Vivo Analyses Reveal a Complex Pharmacogenomic Landscape in Lung Adenocarcinoma. Cancer Res.

[28] Dijkstra KK, Vendramin R, Karagianni D, Witsen M, Gálvez-Cancino F, Hill MS (2025). Subclonal immune evasion in non-small cell lung cancer. Cancer Cell.

[29] Blanchard Z, Brown EA, Ghazaryan A, Welm AL (2025). PDX models for functional precision oncology and discovery science. Nat Rev Cancer.

[30] Lau SCM, Pan Y, Velcheti V, Wong KK (2022). Squamous cell lung cancer: Current landscape and future therapeutic options. Cancer Cell.

[31] Hanahan D (2022). Hallmarks of Cancer: New Dimensions. Cancer Discov.

[32] LaFave LM, Kartha VK, Ma S, Meli K, Del Priore I, Lareau C (2020). Epigenomic State Transitions Characterize Tumor Progression in Mouse Lung Adenocarcinoma. Cancer Cell.

[33] Corces MR, Granja JM, Shams S, Louie BH, Seoane JA, Zhou W (2018). The chromatin accessibility landscape of primary human cancers. Science.

[34] Geffen Y, Anand S, Akiyama Y, Yaron TM, Song Y, Johnson JL (2023). Pan-cancer analysis of post-translational modifications reveals shared patterns of protein regulation. Cell.

[35] Liang W-W, Lu RJ-H, Jayasinghe RG, Foltz SM, Porta-Pardo E, Geffen Y (2023). Integrative multi-omic cancer profiling reveals DNA methylation patterns associated with therapeutic vulnerability and cell-of-origin. Cancer Cell.

[36] Vavilis T, Petre ML, Vatsellas G, Ainatzoglou A, Stamoula E, Sachinidis A (2024). Lung Cancer Proteogenomics: Shaping the Future of Clinical Investigation. Cancers (Basel).

[37] Guo T, Steen JA, Mann M (2025). Mass-spectrometry-based proteomics: from single cells to clinical applications. Nature.

[38] Lundberg E, Borner GHH (2019). Spatial proteomics: a powerful discovery tool for cell biology. Nat Rev Mol Cell Biol.

[39] El-Khoury V, Schritz A, Kim S-Y, Lesur A, Sertamo K, Bernardin F (2020). Identification of a Blood-Based Protein Biomarker Panel for Lung Cancer Detection. Cancers (Basel).

[40] Carrot-Zhang J, Yao X, Devarakonda S, Deshpande A, Damrauer JS, Silva TC (2021). Whole-genome characterization of lung adenocarcinomas lacking the RTK/RAS/RAF pathway. Cell Rep.

[41] Liu L, Zhang J, Wood S, Newell F, Leonard C, Koufariotis LT (2024). Performance of somatic structural variant calling in lung cancer using Oxford Nanopore sequencing technology. BMC Genomics.

[42] Li Y, Liu Y, Xie Y, Wang Y, Wang J, Wang H (2025). Long-read RNA sequencing enables full-length chimeric transcript annotation of transposable elements in lung adenocarcinoma. BMC Cancer.

[43] Lengel HB, Mastrogiacomo B, Connolly JG, Tan KS, Liu Y, Fick CN (2023). Genomic mapping of metastatic organotropism in lung adenocarcinoma. Cancer Cell.

[44] Maynard A, McCoach CE, Rotow JK, Harris L, Haderk F, Kerr DL (2020). Therapy-Induced Evolution of Human Lung Cancer Revealed by Single-Cell RNA Sequencing. Cell.

[45] Facchinetti F, Proto C, Minari R, Garassino M, Tiseo M (2018). Mechanisms of Resistance to Target Therapies in Non-small Cell Lung Cancer. Handb Exp Pharmacol.

[46] Singhal A, Li BT, O’Reilly EM (2024). Targeting KRAS in cancer. Nat Med.

[47] Passaro A, Jänne PA, Mok T, Peters S (2021). Overcoming therapy resistance in EGFR-mutant lung cancer. Nat Cancer.

[48] Quintanal-Villalonga Á, Chan JM, Yu HA, Pe’er D, Sawyers CL, Sen T (2020). Lineage plasticity in cancer: a shared pathway of therapeutic resistance. Nat Rev Clin Oncol.

[49] Awad MM, Liu S, Rybkin II, Arbour KC, Dilly J, Zhu VW (2021). Acquired Resistance to KRASG12C Inhibition in Cancer. N Engl J Med.

[50] Sequist LV, Waltman BA, Dias-Santagata D, Digumarthy S, Turke AB, Fidias P (2011). Genotypic and histological evolution of lung cancers acquiring resistance to EGFR inhibitors. Sci Transl Med.

[51] Zakowski MF, Ladanyi M, Kris MG, Memorial Sloan-Kettering Cancer Center Lung Cancer OncoGenome Group (2006). EGFR mutations in small-cell lung cancers in patients who have never smoked. N Engl J Med.

[52] Mugarza E, van Maldegem F, Boumelha J, Moore C, Rana S, Llorian Sopena M (2022). Therapeutic KRASG12C inhibition drives effective interferon-mediated antitumor immunity in immunogenic lung cancers. Sci Adv.

[53] Nokin M-J, Mira A, Patrucco E, Ricciuti B, Cousin S, Soubeyran I (2024). RAS-ON inhibition overcomes clinical resistance to KRAS G12C-OFF covalent blockade. Nat Commun.

[54] Riedl JM, Matsubara H, McNeil R, Patel PS, Fece de la Cruz F, Gulhan DC (2026). Emerging landscape of KRAS inhibitors in cancer treatment. Cancer Cell.

[55] Araujo HA, Pechuan-Jorge X, Zhou T, Do MT, Hu X, Rojas Alvarez FR (2024). Mechanisms of Response and Tolerance to Active RAS Inhibition in KRAS-Mutant Non-Small Cell Lung Cancer. Cancer Discov.

[56] Wei X, Blaj C, Al-Radhawi Ali, Lai LP, Maldonato BJ, Yang YC (2026). Abrogation of Oncogenic RAS Signaling by a RAS(ON) Inhibitor Doublet Primes Immune-refractory KRAS G12C-mutant NSCLC for Immune Checkpoint Blockade. Cancer Discov.

[57] Russo M, Chen M, Mariella E, Peng H, Rehman SK, Sancho E (2024). Cancer drug-tolerant persister cells: from biological questions to clinical opportunities. Nat Rev Cancer.

[58] Guler GD, Tindell CA, Pitti R, Wilson C, Nichols K, Cheung KaiWai (2017). Repression of Stress-Induced LINE-1 Expression Protects Cancer Cell Subpopulations from Lethal Drug Exposure. Cancer Cell.

[59] Sharma SV, Lee DY, Li B, Quinlan MP, Takahashi F, Maheswaran S (2010). A chromatin-mediated reversible drug-tolerant state in cancer cell subpopulations. Cell.

[60] Baretti M, Danilova L, Durham JN, Betts CB, Cope L, Sidiropoulos DN (2024). Entinostat in combination with nivolumab in metastatic pancreatic ductal adenocarcinoma: a phase 2 clinical trial. Nat Commun.

[61] Roussos Torres ET, Rafie C, Wang C, Lim D, Brufsky A, LoRusso P (2021). Phase I Study of Entinostat and Nivolumab with or without Ipilimumab in Advanced Solid Tumors (ETCTN-9844). Clin Cancer Res.

[62] Sidiropoulos DN, Rafie CI, Jang JK, Castanon S, Baugh AG, Gonzalez E (2022). Entinostat Decreases Immune Suppression to Promote Antitumor Responses in a HER2+ Breast Tumor Microenvironment. Cancer Immunol Res.

[63] Roussos Torres ET, Ho WJ, Danilova L, Tandurella JA, Leatherman J, Rafie C (2024). Entinostat, nivolumab and ipilimumab for women with advanced HER2-negative breast cancer: a phase Ib trial. Nat Cancer.

[64] Oren Y, Tsabar M, Cuoco MS, Amir-Zilberstein L, Cabanos HF, Hütter J-C (2021). Cycling cancer persister cells arise from lineages with distinct programs. Nature.

[65] Mikubo M, Inoue Y, Liu G, Tsao M-S (2021). Mechanism of Drug Tolerant Persister Cancer Cells: The Landscape and Clinical Implication for Therapy. J Thorac Oncol.

[66] Nie M, Hu Z (2024). Metabolic orchestration of drug-tolerant persister cells in cancer. Life medicine.

[67] Li Z, Zhuang X, Pan C-H, Yan Y, Thummalapalli R, Hallin J (2024). Alveolar Differentiation Drives Resistance to KRAS Inhibition in Lung Adenocarcinoma. Cancer Discov.

[68] Liu S, Jiang A, Tang F, Duan M, Li B (2025). Drug-induced tolerant persisters in tumor: mechanism, vulnerability and perspective implication for clinical treatment. Mol Cancer.

[69] Yang M, Cui M, Sun Y, Liu S, Jiang W (2024). Mechanisms, combination therapy, and biomarkers in cancer immunotherapy resistance. Cell Commun Signal.

[70] Wang Z, Elbanna Y, Godet I, Gan S, Peters L, Lampe G (2026). TGFβ induces an atypical EMT to evade immune mechanosurveillance in lung adenocarcinoma dormant metastasis. Nat Cancer.

[71] Hu J, Sánchez-Rivera FJ, Wang Z, Johnson GN, Ho Y-J, Ganesh K (2023). STING inhibits the reactivation of dormant metastasis in lung adenocarcinoma. Nature.

[72] Abbosh C, Hodgson D, Doherty GJ, Gale D, Black JRM, Horn L (2024). Implementing circulating tumor DNA as a prognostic biomarker in resectable non-small cell lung cancer. Trends Cancer.

[73] Chabon JJ, Hamilton EG, Kurtz DM, Esfahani MS, Moding EJ, Stehr H (2020). Integrating genomic features for non-invasive early lung cancer detection. Nature.

[74] Black JRM, Karasaki T, Abbott CW, Li B, Veeriah S, Al Bakir M (2025). Longitudinal ultrasensitive ctDNA monitoring for high-resolution lung cancer risk prediction. Cell.

[75] Black JRM, Bartha G, Abbott CW, Boyle SM, Karasaki T, Li B (2025). Ultrasensitive ctDNA detection for preoperative disease stratification in early-stage lung adenocarcinoma. Nat Med.

[76] Gale D, Heider K, Ruiz-Valdepenas A, Hackinger S, Perry M, Marsico G (2022). Residual ctDNA after treatment predicts early relapse in patients with early-stage non-small cell lung cancer. Ann Oncol.

[77] Herbst RS, John T, Grohé C, Goldman JW, Kato T, Laktionov K (2025). Molecular residual disease analysis of adjuvant osimertinib in resected EGFR-mutated stage IB–IIIA non-small-cell lung cancer. Nat Med.

[78] Moding EJ, Nabet BY, Alizadeh AA, Diehn M (2021). Detecting Liquid Remnants of Solid Tumors: Circulating Tumor DNA Minimal Residual Disease. Cancer Discov.

[79] de Souza N, Zhao S, Bodenmiller B (2024). Multiplex protein imaging in tumour biology. Nat Rev Cancer.

[80] Tan AC, Tan DSW (2022). Targeted Therapies for Lung Cancer Patients With Oncogenic Driver Molecular Alterations. J Clin Oncol.

[81] Mullard A (2023). Chemoproteomic trailblazer advances covalent candidate into the clinic, pushes allosteric agenda. Nat Rev Drug Discov.

[82] Heymach JV, Ruiter G, Ahn M-J, Girard N, Smit EF, Planchard D (2025). Zongertinib in Previously Treated HER2-Mutant Non-Small-Cell Lung Cancer. N Engl J Med.

[83] Wilding B, Woelflingseder L, Baum A, Chylinski K, Vainorius G, Gibson N (2025). Zongertinib (BI 1810631), an Irreversible HER2 TKI, Spares EGFR Signaling and Improves Therapeutic Response in Preclinical Models and Patients with HER2-Driven Cancers. Cancer Discov.

[84] Ebright RY, Dilly J, Shaw AT, Aguirre AJ (2025). Response and Resistance to RAS Inhibition in Cancer. Cancer Discov.

[85] Hofmann MH, Gerlach D, Misale S, Petronczki M, Kraut N (2022). Expanding the Reach of Precision Oncology by Drugging All KRAS Mutants. Cancer Discov.

[86] Le X, Kim TM, Loong HH, Prelaj A, Goh BC, Li L (2025). Sevabertinib in Advanced HER2-Mutant Non-Small-Cell Lung Cancer. N Engl J Med.

[87] Siegel F, Siegel S, Kotýnková K, Karsli Uzunbas G, Korr D, Tomono H (2025). Sevabertinib, a Reversible HER2 Inhibitor with Activity in Lung Cancer. Cancer Discov.

[88] Repetto M, Fernandez N, Drilon A, Chakravarty D (2024). Precision Oncology: 2024 in Review. Cancer Discov.

[89] Belmontes B, Slemmons KK, Su C, Liu S, Policheni AN, Moriguchi J (2025). AMG 193, a Clinical Stage MTA-Cooperative PRMT5 Inhibitor, Drives Antitumor Activity Preclinically and in Patients with MTAP-Deleted Cancers. Cancer Discov.

[90] Zanchetta C, De Marchi L, Macerelli M, Pelizzari G, Costa J, Aprile G (2024). Antibody-Drug Conjugates in Non-Small Cell Lung Cancer: State of the Art and Future Perspectives. Int J Mol Sci.

[91] Colombo R, Tarantino P, Rich JR, LoRusso PM, de Vries EGE (2024). The Journey of Antibody-Drug Conjugates: Lessons Learned from 40 Years of Development. Cancer Discov.

[92] Mountzios G, Sun L, Cho BC, Demirci U, Baka S, Gümüş M (2025). Tarlatamab in Small-Cell Lung Cancer after Platinum-Based Chemotherapy. N Engl J Med.

[93] Rudin CM, Reck M, Johnson ML, Blackhall F, Hann CL, Yang JC-H (2023). Emerging therapies targeting the delta-like ligand 3 (DLL3) in small cell lung cancer. J Hematol Oncol.

[94] Rudin CM, Balli D, Lai WV, Richards AL, Nguyen E, Egger JV (2023). Clinical Benefit From Immunotherapy in Patients With SCLC Is Associated With Tumor Capacity for Antigen Presentation. J Thorac Oncol.

[95] Kang K, Liu S, Yao Z, Xue J, Lu Y (2025). Addressing clinical needs in NSCLC immunotherapy: Mechanisms of resistance and promising combination strategies. Cell Rep Med.

[96] Jiang J, Yuan J, Hu Z, Zhang Y, Zhang T, Xu M (2022). Systematic illumination of druggable genes in cancer genomes. Cell Rep.

[97] Stuart DD, Guzman-Perez A, Brooijmans N, Jackson EL, Kryukov GV, Friedman AA (2023). Precision Oncology Comes of Age: Designing Best-in-Class Small Molecules by Integrating Two Decades of Advances in Chemistry, Target Biology, and Data Science. Cancer Discov.

[98] Hinterndorfer M, Spiteri VA, Ciulli A, Winter GE (2025). Targeted protein degradation for cancer therapy. Nat Rev Cancer.

[99] Mullard A (2024). Protein degraders push into novel target space. Nat Rev Drug Discov.

[100] Moll HP, Pranz K, Musteanu M, Grabner B, Hruschka N, Mohrherr J (2018). Afatinib restrains K-RAS-driven lung tumorigenesis. Sci Transl Med.

[101] Manchado E, Weissmueller S, Morris JP, Chen C-C, Wullenkord R, Lujambio A (2016). A combinatorial strategy for treating KRAS-mutant lung cancer. Nature.

[102] Rudin CM, Brambilla E, Faivre-Finn C, Sage J (2021). Small-cell lung cancer. Nat Rev Dis Primers.

[103] Dhillon S (2024). Tarlatamab: First Approval. Drugs.

[104] Torchia A, Ciappina G, Giammaruco M, Monteferrante I, Landi L, Cappuzzo F (2025). Antibody-Based Therapeutics in Small Cell Lung Cancer: A Narrative Review. Biologics.

[105] Owonikoko TK, Niu H, Nackaerts K, Csoszi T, Ostoros G, Mark Z (2020). Randomized Phase II Study of Paclitaxel plus Alisertib versus Paclitaxel plus Placebo as Second-Line Therapy for SCLC: Primary and Correlative Biomarker Analyses. J Thorac Oncol.

[106] Puzio-Kuter AM, Xu L, McBrayer MK, Dominique R, Li HH, Fahr BJ (2025). Restoration of the Tumor Suppressor Function of Y220C-Mutant p53 by Rezatapopt, a Small-Molecule Reactivator. Cancer Discov.

[107] Rudin CM, Poirier JT, Byers LA, Dive C, Dowlati A, George J (2019). Molecular subtypes of small cell lung cancer: a synthesis of human and mouse model data. Nat Rev Cancer.

[108] Simpson KL, Rothwell DG, Blackhall F, Dive C (2025). Challenges of small cell lung cancer heterogeneity and phenotypic plasticity. Nat Rev Cancer.

[109] Yap TA, Kwiatkowski DJ, Dagogo-Jack I, Offin M, Zauderer MG, Kratzke R (2025). YAP/TEAD inhibitor VT3989 in solid tumors: a phase 1/2 trial. Nat Med.

[110] Sen T, Dotsu Y, Corbett V, Puri S, Sen U, Boyle TA (2025). Pulmonary neuroendocrine neoplasms: the molecular landscape, therapeutic challenges, and diagnosis and management strategies. Lancet Oncol.

[111] Shields MD, Minton KG, Tran M, Gunderman PR, Larsson LG, Guo S (2025). Defining the needle in a haystack: A compendium of genomic, pathologic, and clinical characteristics of rare pulmonary tumors. Lung Cancer.

[112] Choudhury NJ, Lavery JA, Brown S, de Bruijn I, Jee J, Tran TN (2023). The GENIE BPC NSCLC Cohort: A Real-World Repository Integrating Standardized Clinical and Genomic Data for 1,846 Patients with Non-Small Cell Lung Cancer. Clin Cancer Res.

[113] Feng X, Shu W, Li M, Li J, Xu J, He M (2024). Pathogenomics for accurate diagnosis, treatment, prognosis of oncology: a cutting edge overview. J Transl Med.

[114] Jee J, Fong C, Pichotta K, Tran TN, Luthra A, Waters M (2024). Automated real-world data integration improves cancer outcome prediction. Nature.

[115] Subramanian J, Govindan R (2010). Lung cancer in “Never-smokers”: a unique entity. Oncology (Williston Park).

[116] Tian Y, Ma R, Zhao W, Wang S, Zhou C, Wu W (2025). Comprehensive characterization of early-onset lung cancer, in Chinese young adults. Nat Commun.

[117] Ruiz R, Galvez-Nino M, Roque K, Montes J, Nuñez M, Raez L (2022). Genomic landscape of lung cancer in the young. Front Oncol.

[118] Devarakonda S, Li Y, Martins Rodrigues F, Sankararaman S, Kadara H, Goparaju C (2021). Genomic Profiling of Lung Adenocarcinoma in Never-Smokers. J Clin Oncol.

[119] Zhuang X, Wang Q, Joost S, Ferrena A, Humphreys DT, Li Z (2025). Ageing limits stemness and tumorigenesis by reprogramming iron homeostasis. Nature.

[120] Ernster VL (1994). The epidemiology of lung cancer in women. Ann Epidemiol.

[121] Siegfried JM (2001). Women and lung cancer: does oestrogen play a role?. Lancet Oncol.

[122] Viñolas NN, Garcia-Campelo R, Majem M, Carcereny E, Isla D, Gonzalez-Larriba JL (2020). Assessment of the psychosocial and economic impact according to sex in non-small cell lung cancer patients: an exploratory longitudinal study. BMC Psychol.

[123] Shah SK, Krishnan V, Khan AA, Fass L, Chaudhry T, Seder CW (2024). Women are Underrepresented in Non-small Cell Lung Cancer Clinical Trials: A Systematic Review. Ann Surg Oncol.

[124] Florez N, Kiel L, Riano I, Patel S, DeCarli K, Dhawan N (2024). Lung Cancer in Women: The Past, Present, and Future. Clin Lung Cancer.

[125] Sun S, Schiller JH, Gazdar AF (2007). Lung cancer in never smokers--a different disease. Nat Rev Cancer.

[126] Thompson CA, Gomez SL, Hastings KG, Kapphahn K, Yu P, Shariff-Marco S (2016). The Burden of Cancer in Asian Americans: A Report of National Mortality Trends by Asian Ethnicity. Cancer Epidemiol Biomarkers Prev.

[127] Zhu DT, Lai A, Park A, Zhong A, Tamang S (2025). Disparities in Cancer Mortality among Disaggregated Asian American Subpopulations, 2018-2021. J Racial Ethn Health Disparities.

[128] Gomez SL, DeRouen M, Chen MS, Wakelee H, Velotta JB, Sakoda LC (2025). Elevated risk of lung cancer among Asian American women who have never smoked: an emerging cancer disparity. J Natl Cancer Inst.

[129] Zhang T, Joubert P, Ansari-Pour N, Zhao W, Hoang PH, Lokanga R (2021). Genomic and evolutionary classification of lung cancer in never smokers. Nat Genet.

[130] Díaz-Gay M, Zhang T, Hoang PH, Leduc C, Baine MK, Travis WD (2025). The mutagenic forces shaping the genomes of lung cancer in never smokers. Nature.

[131] LoPiccolo J, Gusev A, Christiani DC, Jänne PA (2024). Lung cancer in patients who have never smoked - an emerging disease. Nat Rev Clin Oncol.

[132] Herbst RS, Gandara DR, Hirsch FR, Redman MW, LeBlanc M, Mack PC (2015). Lung Master Protocol (Lung-MAP)-A Biomarker-Driven Protocol for Accelerating Development of Therapies for Squamous Cell Lung Cancer: SWOG S1400. Clin Cancer Res.

[133] Herbst RS, Blanke CD, Sigal EV (2024). Novel Approach to Accelerate Lung Cancer Research: Lung-MAP and the Potential of Public-Private Partnerships. Clin Cancer Res.

[134] Dragnev KH, Redman MW, Reckamp KL, Khalil M, Henick BS, Moon J (2025). PRAGMATICA-LUNG (SWOG S2302): A prospective, randomized study of ramucirumab plus pembrolizumab versus standard of care for participants previously treated with immunotherapy for stage IV or recurrent non-small cell lung cancer. Journal of Clinical Oncology.

[135] Liu S-YM, Tu H-Y, Wei X-W, Yan H-H, Dong X-R, Cui J-W (2023). First-line pyrotinib in advanced HER2-mutant non-small-cell lung cancer: a patient-centric phase 2 trial. Nat Med.

[136] Bi J-H, Tuo J-Y, Xiao Y-X, Tang D-D, Zhou X-H, Jiang Y-F (2024). Observed and relative survival trends of lung cancer: A systematic review of population-based cancer registration data. Thorac Cancer.

[137] Bonney A, Malouf R, Marchal C, Manners D, Fong KM, Marshall HM (2022). Impact of low-dose computed tomography (LDCT) screening on lung cancer-related mortality. Cochrane Database Syst Rev.

[138] Moyer VA, US. Preventive Services Task Force (2014). Screening for lung cancer: U.S. Preventive Services Task Force recommendation statement. Ann Intern Med.

[139] Kratzer TB, Bandi P, Freedman ND, Smith RA, Travis WD, Jemal A (2024). Lung cancer statistics, 2023. Cancer.

[140] Krist AH, Davidson KW, Mangione CM, Barry MJ, Cabana M, US Preventive Services Task Force (2021). Screening for Lung Cancer: US Preventive Services Task Force Recommendation Statement. JAMA.

[141] Ostrin EJ, Sidransky D, Spira A, Hanash SM (2020). Biomarkers for Lung Cancer Screening and Detection. Cancer Epidemiol Biomarkers Prev.

[142] Christensen J, Prosper AE, Wu CC, Chung J, Lee E, Elicker B (2024). ACR Lung-RADS v2022: Assessment Categories and Management Recommendations. Chest.

[143] Zhu E, Muneer A, Zhang J, Xia Y, Li X, Zhou C (2025). Progress and challenges of artificial intelligence in lung cancer clinical translation. NPJ Precis Oncol.

[144] Lastwika KJ, Wu W, Zhang Y, Ma N, Zečević M, Pipavath SNJ (2023). Multi-Omic Biomarkers Improve Indeterminate Pulmonary Nodule Malignancy Risk Assessment. Cancers (Basel).

[145] Jeon J, Holford TR, Levy DT, Feuer EJ, Cao P, Tam J (2018). Smoking and Lung Cancer Mortality in the United States From 2015 to 2065: A Comparative Modeling Approach. Ann Intern Med.

[146] Kerpel-Fronius A, Tammemägi M, Cavic M, Henschke C, Jiang L, Kazerooni E (2022). Screening for Lung Cancer in Individuals Who Never Smoked: An International Association for the Study of Lung Cancer Early Detection and Screening Committee Report. J Thorac Oncol.

[147] Berg CD, Schiller JH, Boffetta P, Cai J, Connolly C, Kerpel-Fronius A (2023). Air Pollution and Lung Cancer: A Review by International Association for the Study of Lung Cancer Early Detection and Screening Committee. J Thorac Oncol.

[148] Wu JT-Y, Wakelee HA, Han SS (2023). Optimizing Lung Cancer Screening With Risk Prediction: Current Challenges and the Emerging Role of Biomarkers. J Clin Oncol.

[149] Chang G-C, Chiu C-H, Yu C-J, Chang Y-C, Chang Y-H, Hsu K-H (2024). Low-dose CT screening among never-smokers with or without a family history of lung cancer in Taiwan: a prospective cohort study. Lancet Respir Med.

[150] Lai G, Ooi GSK, Too CW, Huang M, Chia CML, Saw SPL (2023). EP04.05-05 Singapore Lung Cancer Screening through Integrating CT with Other Biomarkers (SOLSTICE), A Single Arm Lung Cancer Screening Study. Journal of Thoracic Oncology.

[151] Feng X, Zahed H, Onwuka J, Callister MEJ, Johansson M, Etzioni R (2024). Cancer Stage Compared With Mortality as End Points in Randomized Clinical Trials of Cancer Screening: A Systematic Review and Meta-Analysis. JAMA.

[152] Tian R, Wiley B, Liu J, Zong X, Truong B, Zhao S (2023). Clonal Hematopoiesis and Risk of Incident Lung Cancer. J Clin Oncol.

[153] Park MD, Le Berichel J, Hamon P, Wilk CM, Belabed M, Yatim N (2024). Hematopoietic aging promotes cancer by fueling IL-1α–driven emergency myelopoiesis. Science (1979).

[154] Ridker PM, MacFadyen JG, Thuren T, Everett BM, Libby P, Glynn RJ (2017). Effect of interleukin-1β inhibition with canakinumab on incident lung cancer in patients with atherosclerosis: exploratory results from a randomised, double-blind, placebo-controlled trial. Lancet.

[155] Abbate A, Toldo S, Marchetti C, Kron J, Van Tassell BW, Dinarello CA (2020). Interleukin-1 and the Inflammasome as Therapeutic Targets in Cardiovascular Disease. Circ Res.

[156] Al Bakir M, Reading JL, Gamble S, Rosenthal R, Uddin I, Rowan A (2025). Clonal driver neoantigen loss under EGFR TKI and immune selection pressures. Nature.

[157] Pazoki A, Dadfar S, Alirezaee A, Oksenych V, Haghmorad D (2025). Lung cancer vaccine strategies: exploring the spectrum from traditional to RNA-based platforms. Front Bioeng Biotechnol.

[158] Herbst RS (2026). Navigating the Evolving Landscape of EGFR-Mutated NSCLC. N Engl J Med.

